# Variant Impact Predictor database (VIPdb), version 2: trends from three decades of genetic variant impact predictors

**DOI:** 10.1186/s40246-024-00663-z

**Published:** 2024-08-28

**Authors:** Yu-Jen Lin, Arul S. Menon, Zhiqiang Hu, Steven E. Brenner

**Affiliations:** 1 Department of Molecular and Cell Biology, University of California, Berkeley, CA 94720 USA; 2 Center for Computational Biology, University of California, Berkeley, CA 94720 USA; 3 College of Computing, Data Science, and Society, University of California, Berkeley, CA 94720 USA; 4 Department of Plant and Microbial Biology, University of California, 111 Koshland Hall #3102, Berkeley, CA 94720-3102 USA; 5 Present Address: Illumina, Foster City, CA 94404 USA

**Keywords:** VIPdb, Variant Impact Predictor (VIP), Variant Effect Predictor (VEP), Genomic variant, Variant interpretation, SNV, SV, Indel, Genotype–phenotype relationship

## Abstract

**Background:**

Variant interpretation is essential for identifying patients’ disease-causing genetic variants amongst the millions detected in their genomes. Hundreds of Variant Impact Predictors (VIPs), also known as Variant Effect Predictors (VEPs), have been developed for this purpose, with a variety of methodologies and goals. To facilitate the exploration of available VIP options, we have created the Variant Impact Predictor database (VIPdb).

**Results:**

The Variant Impact Predictor database (VIPdb) version 2 presents a collection of VIPs developed over the past three decades, summarizing their characteristics, ClinGen calibrated scores, CAGI assessment results, publication details, access information, and citation patterns. We previously summarized 217 VIPs and their features in VIPdb in 2019. Building upon this foundation, we identified and categorized an additional 190 VIPs, resulting in a total of 407 VIPs in VIPdb version 2. The majority of the VIPs have the capacity to predict the impacts of single nucleotide variants and nonsynonymous variants. More VIPs tailored to predict the impacts of insertions and deletions have been developed since the 2010s. In contrast, relatively few VIPs are dedicated to the prediction of splicing, structural, synonymous, and regulatory variants. The increasing rate of citations to VIPs reflects the ongoing growth in their use, and the evolving trends in citations reveal development in the field and individual methods.

**Conclusions:**

VIPdb version 2 summarizes 407 VIPs and their features, potentially facilitating VIP exploration for various variant interpretation applications. VIPdb is available at https://genomeinterpretation.org/vipdb

**Supplementary Information:**

The online version contains supplementary material available at 10.1186/s40246-024-00663-z.

## Background

Advances in sequencing technologies, including gene panels, whole exome sequencing, whole genome sequencing, and long read sequencing, have revolutionized the investigation of genetic variation on a large scale and hence have accelerated the discovery of novel genetic etiologies of diseases and improved the efficiency of diagnosis [1, 2]. Typically, thousands to millions of variants are identified in each individual [3, 4], making it challenging to distinguish disease-causing variants from non-contributory ones. Consequently, methods to predict the impacts of variants being disease-causing are essential [5, 6].

This need prompted the development of Variant Impact Predictors (VIPs), tools or databases designed to predict the consequences of genetic variants. The first VIP (known to us) was developed in 1993 to predict different types of collagen variants involved in osteogenesis imperfecta, using decision trees [7]. Since then, hundreds of genetic VIPs have been developed, with a variety of methodologies and goals [8]. Some overlapping categories of variants considered by different tools are single nucleotide variations (SNVs), insertions and deletions (indels), structural variations (SVs), nonsynonymous variants, synonymous variants, splicing variants, and regulatory variants. VIPs are designed for different contexts, such as for germline variants, somatic variants, or specific diseases or genes. While most provide pathogenicity scores, some provide valuable information about molecular mechanisms and other details [9]. The variety of VIPs underscores the complex nature of variant interpretation and poses a challenge for users in identifying the most suitable VIPs for their specific needs, and VIPdb aims to help support transparency to inform these decisions.

Many computational impact prediction methods have been developed, yet the field lacks a clear consensus on their appropriate use and interpretation [10]. Recognizing the need for an organized approach to explore available VIPs, several research entities have constructed resources facilitating the informed use of VIPs. Initiatives like the Critical Assessment of Genome Interpretation (CAGI) conduct community experiments to assess VIPs across different variant types and contexts (https://genomeinterpretation.org) [10–12]. The dbNSFP (database for Nonsynonymous Single-nucleotide polymorphisms’ Functional Predictions) hosts precomputes of several VIP results [13]. OpenCRAVAT integrates hundreds of VIP analyses of cancer-related variants in one platform, enhancing accessibility for users [14]. These resources have played an important role in introducing users to VIP options. Consequently, we developed VIPdb to serve as a comprehensive resource for exploring VIPs.

To systematically evaluate the pathogenicity of a variant in a clinical laboratory, ACMG/AMP has established guidelines for interpreting genetic variants that integrate several lines of evidence, including population data, functional data, segregation data, and computational prediction [15]. ClinGen, CGC, and VICC also have developed standards for the classification of pathogenicity of somatic variants in cancer [16]. Historically, VIPs provided only supporting evidence in determining the pathogenicity or benignity of variants in clinical settings. However, recent ClinGen clinical recommendations allow VIPs the potential to provide stronger evidence [17]. This greater role for VIPs in providing evidence for clinical decisions could improve genetic disease diagnosis.

The Variant Impact Predictor database (VIPdb) offers a curation of available computational tools for predicting variant impact. Initially established in 2007 and 2010 [18], the database was last updated in 2019 [8]. VIPdb version 2 is a comprehensive update through January 2, 2024, with select additional methods added through July 2024 (Supplementary Table S1).

## Implementation

Our identification of VIPs involved searching for potential VIPs and examining their articles to determine whether they should be included in VIPdb. In the initial step, we searched the literature using the query “(((tool(Title]) OR (pipeline(Title])) AND (variant(Title/Abstract]))” on PubMed and collected potential VIPs citing pioneering VIPs (SIFT, PolyPhen, ANNOVAR, and SnpEff) [19–30]. Additionally, we gathered potential VIPs from existing databases such as OpenCRAVAT and dbNSFP, as well as from submissions by VIP developers. Subsequently, we examined the literature and included only programs capable of handling variant data, such as VCF files, rsID, or location in the genome, and providing evidence or predictions of the variant impacts. Overall, this resulted in the identification of 190 additional VIPs, augmenting the VIPdb to a total of 407 VIPs (Supplementary Table S1) [7, 13, 19–421].

To facilitate users’ exploration of available VIPs, we described key features of each VIP. VIPs primarily designed for variant impact prediction were labeled as such. VIPs not originally designed for variant impact prediction but nonetheless used for this purpose, such as those estimating conservation scores and population allele frequencies, were categorized as non-primary. VIPs that consist of data collected from elsewhere, such as clinical classifications and functional data, were categorized as databases. Conversely, VIPs that compute variant impact predictions were classified as computational tools (labeled as non-databases) even if the data available are precomputed by the tool. Furthermore, as VIPs are designed for different types of genetic variants, we classified the VIPs according to the following overlapping categories of input: single nucleotide variant (SNV), insertion and deletion (indel) variant, structural variant (SV), nonsynonymous/nonsense variant, synonymous variant, splicing variant, and regulatory region variants, with some overlap among these categories. Licensing information, including whether the VIP is free for academic or commercial use, was also included. In addition, we provided details about accessing VIPs, such as homepage links and source code availability.

In VIPdb version 2, we have made enhancements to inform clinical decision-making. We incorporated calibrated threshold scores recommended by ClinGen for clinical use [17] with ACMG/AMP guidelines for variant classification [15]. Additionally, we included community assessment results from the CAGI 6 Annotate All Missense / Missense Marathon challenge [422] to enable users to compare the overall performance of methods and the performance on subsets with high specificity or high sensitivity.

To understand the trends of genetic VIPs over the past three decades, we conducted a citation analysis. We utilized the Entrez module in Biopython to retrieve citation information from the PubMed database. Specifically, the elink function was employed to collect the number of articles citing each VIP, and the esummary function allowed for the collection of publication years for these citations. These functions facilitated the automatic collection of citation numbers by year for each VIP.

In summary, VIPdb version 2 presents a collection of 407 VIPs developed over the past three decades, with their characteristics, citation patterns, publication details, and access information (Supplementary Table S1). VIPdb version 2 is publicly accessible at https://genomeinterpretation.org/vipdb and can be downloaded as a comma-separated values table (Supplementary Table S1).

## Results

We incorporated 190 additional VIPs into VIPdb version 2, alongside the existing 217 VIPs in the previous version of VIPdb. The characteristics of the 407 VIPs are listed in Supplementary Table S1. Among the 407 VIPs in VIPdb version 2, 278 are core VIPs, defined as VIPs primarily designed for variant impact prediction and not a database.

**Fig. 1:**
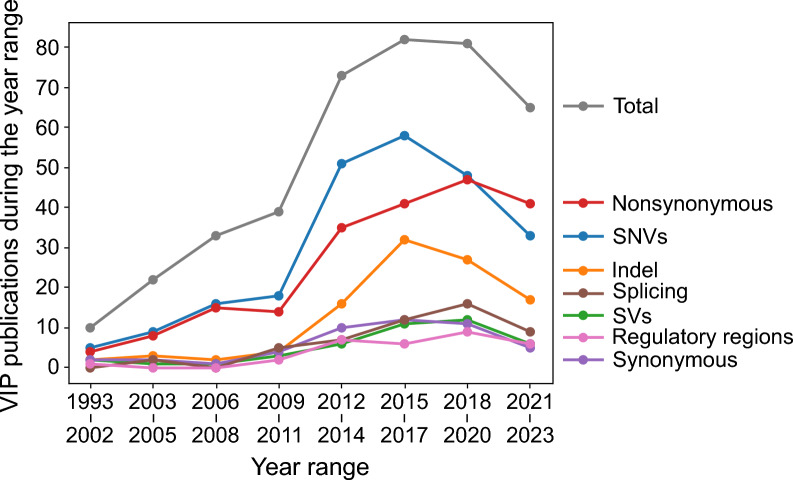
VIP variant type focus

An analysis of the variant type used by VIP showed a predominant focus on predicting the impacts of single nucleotide variants (SNVs) and nonsynonymous variants (Fig. 1). Since the 2010s, there has been a notable surge in the development of VIPs tailored for insertions and deletions (indels), while VIPs dedicated to predicting the impacts of splicing, structural, synonymous, and regulatory variants have grown more modestly (Fig. 1). These observations about VIP variant type not only highlight current focus on but also identify areas that have been less explored, suggesting potential directions for future research.

**Fig. 2:**
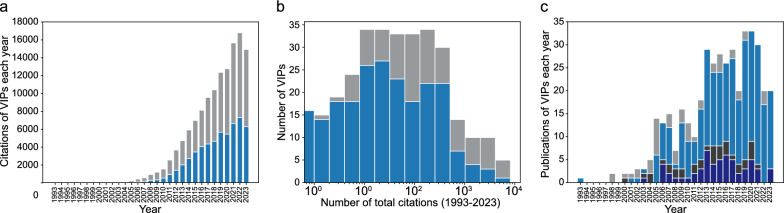
Citation and publication analysis of 407 VIPs. **a** Citations each year for 278 core VIPs (blue) and 129 non-core VIPs (gray). **b** Histogram of total citations for core VIPs (blue) and non-core VIPs (gray). **c** VIPs published per year, with original publications in light blue (core) and light gray (non-core), and subsequent publications in dark blue (core) and dark gray (non-core)

The citation rate of VIPs continues to rise, while the annual publications of VIPs have reached a plateau (Fig. 2). The increasing citation rates for both the 278 core VIPs and the 129 non-core VIPs reflect the ongoing growth of VIP usage (Fig. 2A). The median total citation for VIPs is 41 from 1993 to 2023, with a 95% quantile of 2559 citations (Fig. 2B). Annual publication showed a stabilization in VIP publications, with some being subsequent publications from previous work (Fig. 2C).

**Fig. 3:**
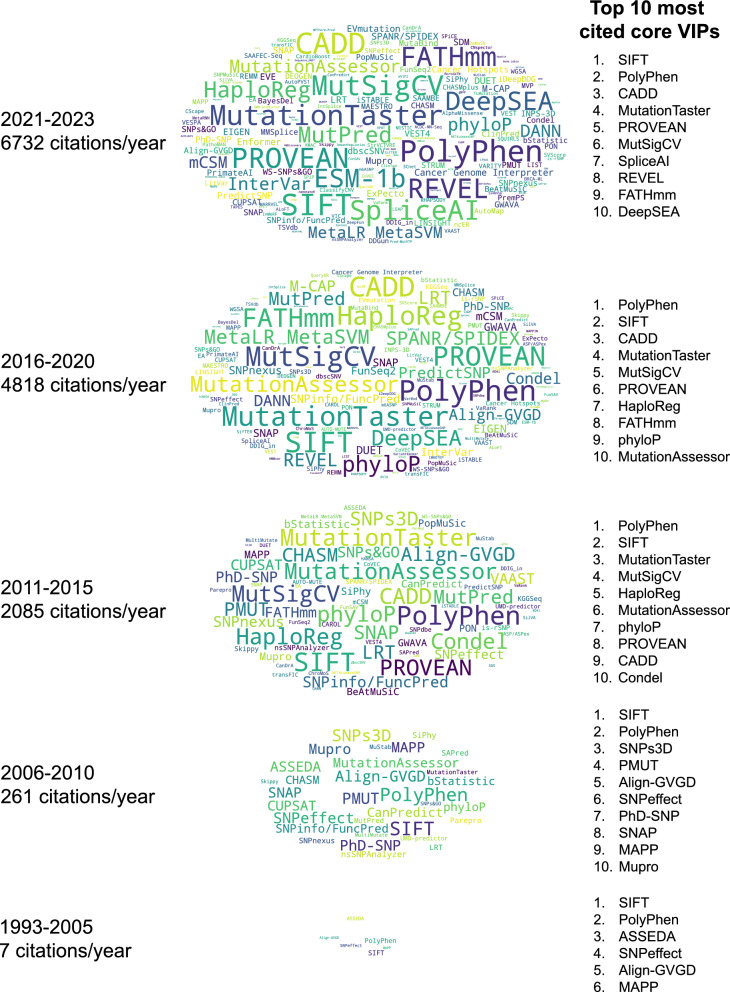
Citation trend of 278 core VIPs (1993–2023). Word clouds representing core VIPs over a specific time period, using cumulative citations for core VIPs with multiple publications. Font sizes in the word clouds correspond to the logarithm of citation counts for each period, and cloud heights are scaled by the logarithm of the annual citation averages. The top 10 most cited core VIPs during the period are listed. *Note*: Core VIPs are methods primarily designed for variant impact prediction and are not classified as databases

**Fig. 4:**
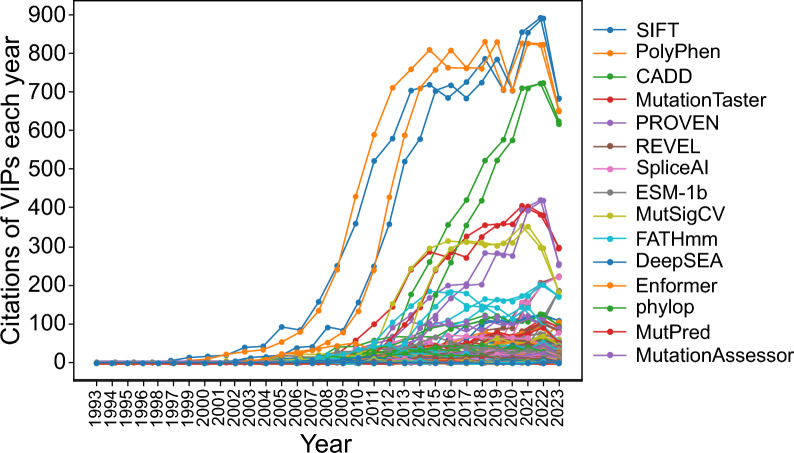
Citation trend of the top 15 most cited core VIPs in the year 2023. *Note*: Core VIPs are methods primarily designed for variant impact prediction and are not classified as databases

The citation trend of 278 core VIPs from 1993 to 2023 is shown in Figs. 3 and 4. The citation analysis revealed that SIFT and PolyPhen, among the earliest genome-wide ones, are the most cited core VIPs (Figs. 3 and 4).

## Discussion and conclusions

VIPdb version 2 provides a comprehensive view of VIPs. To identify the most appropriate VIPs for user’s specific needs, users are advised to thoroughly assess the strengths and weaknesses of VIPs before determining their suitability for use. For example, initiatives like the Critical Assessment of Genome Interpretation (CAGI) conduct community experiments to assess VIPs across different variant types and contexts [10–12].

Beyond adding new methods as they become available, we plan to enhance VIPdb by adding new fields that increase transparency, such as reporting of molecular mechanisms [9]. Additionally, we will incorporate some model information, such as details about the training data, training date, and training method used. New CAGI results and ClinGen calibration will also be added. We welcome suggestions for additional feature fields to be curated in future updates.

With 407 curated VIPs, VIPdb version 2 provides a comprehensive overview of programs designed for variant impact prediction, along with their characteristics, citation patterns, publication details, and access information. VIPdb version 2 is available on the CAGI website (https://genomeinterpretation.org/vipdb) and is also included in Supplementary Table S1. We invite submissions of new VIPs for the next version of VIPdb.

### Supplementary Information

Supplementary Material 1.

Supplementary Material 2.

## Data Availability

Project name: Variant Impact Predictor Database (VIPdb). Project home page: https://genomeinterpretation.org/vipdb. Operating system(s): Platform independent. Programming language: Not applicable. Other requirements: Not applicable. Any restrictions to use by non-academics: Not applicable. VIPdb version 2 is available at https://genomeinterpretation.org/vipdb and is also included in Supplementary Table S1 for this paper.

## Abbreviations

VIP: Variant Impact Predictor
VIPdb: Variant Impact Predictor database

## References

[1] Marwaha S, Knowles JW, Ashley EA. A guide for the diagnosis of rare and undiagnosed disease: beyond the exome. Genome Med. 2022;14(1):23. 35220969 10.1186/s13073-022-01026-w PMC8883622

[2] Schobers G, Derks R, den Ouden A, Swinkels H, van Reeuwijk J, Bosgoed E, et al. Genome sequencing as a generic diagnostic strategy for rare disease. Genome Med. 2024;16(1):32. 38355605 10.1186/s13073-024-01301-y PMC10868087

[3] Fowler DM, Adams DJ, Gloyn AL, Hahn WC, Marks DS, Muffley LA, et al. An Atlas of variant effects to understand the genome at nucleotide resolution. Genome Biol. 2023;24(1):147. 37394429 10.1186/s13059-023-02986-x PMC10316620

[4] Marian AJ. Clinical interpretation and management of genetic variants. JACC Basic Transl Sci. 2020;5(10):1029–42. 33145465 10.1016/j.jacbts.2020.05.013 PMC7591931

[5] Papadimitriou S, Gazzo A, Versbraegen N, Nachtegael C, Aerts J, Moreau Y, et al. Predicting disease-causing variant combinations. Proc Natl Acad Sci U S A. 2019;116(24):11878–87. 31127050 10.1073/pnas.1815601116 PMC6575632

[6] Wang D, Li J, Wang Y, Wang E. A comparison on predicting functional impact of genomic variants. NAR Genom Bioinform. 2022;4(1):lqab122. 35047814 10.1093/nargab/lqab122 PMC8759571

[7] Hunter L, Klein T. Finding relevant biomolecular features. Proc Int Conf Intell Syst Mol Biol. 1993;1:190–7. 7584335

[8] Hu Z, Yu C, Furutsuki M, Andreoletti G, Ly M, Hoskins R, et al. VIPdb, a genetic Variant Impact Predictor database. Hum Mutat. 2019;40(9):1202–14. 31283070 10.1002/humu.23858 PMC7288905

[9] Karchin R, Radivojac P, O’Donnell-Luria A, Greenblatt MS, Tolstorukov MY, Sonkin D. Improving transparency of computational tools for variant effect prediction. Nat Genet. 2024;56(7):1324–6. 38956207 10.1038/s41588-024-01821-8 PMC11330584

[10] Andreoletti G, Pal LR, Moult J, Brenner SE. Reports from the fifth edition of CAGI: the critical assessment of genome interpretation. Hum Mutat. 2019;40(9):1197–201. 31334884 10.1002/humu.23876 PMC7329230

[11] The Critical Assessment of Genome Interpretation Consortium. CAGI, the Critical Assessment of Genome Interpretation, establishes progress and prospects for computational genetic variant interpretation methods. Genome Biol. 2024;25(1):53. 38389099 10.1186/s13059-023-03113-6 PMC10882881

[12] Hoskins RA, Repo S, Barsky D, Andreoletti G, Moult J, Brenner SE. Reports from CAGI: the critical assessment of genome interpretation. Hum Mutat. 2017;38(9):1039–41. 28817245 10.1002/humu.23290 PMC5606199

[13] Liu X, Li C, Mou C, Dong Y, Tu Y. dbNSFP v4: a comprehensive database of transcript-specific functional predictions and annotations for human nonsynonymous and splice-site SNVs. Genome Med. 2020;12(1):103. 33261662 10.1186/s13073-020-00803-9 PMC7709417

[14] Pagel KA, Kim R, Moad K, Busby B, Zheng L, Tokheim C, et al. Integrated informatics analysis of cancer-related variants. JCO Clin Cancer Inform. 2020;4:310–7. 32228266 10.1200/CCI.19.00132 PMC7113103

[15] Richards S, Aziz N, Bale S, Bick D, Das S, Gastier-Foster J, et al. Standards and guidelines for the interpretation of sequence variants: a joint consensus recommendation of the American College of Medical Genetics and Genomics and the Association for Molecular Pathology. Genet Med. 2015;17(5):405–24. 25741868 10.1038/gim.2015.30 PMC4544753

[16] Horak P, Griffith M, Danos AM, Pitel BA, Madhavan S, Liu X, et al. Standards for the classification of pathogenicity of somatic variants in cancer (oncogenicity): Joint recommendations of Clinical Genome Resource (ClinGen), Cancer Genomics Consortium (CGC), and Variant Interpretation for Cancer Consortium (VICC). Genet Med. 2022;24(5):986–98. 35101336 10.1016/j.gim.2022.01.001 PMC9081216

[17] Pejaver V, Byrne AB, Feng BJ, Pagel KA, Mooney SD, Karchin R, et al. Calibration of computational tools for missense variant pathogenicity classification and ClinGen recommendations for PP3/BP4 criteria. Am J Hum Genet. 2022;109(12):2163–77. 36413997 10.1016/j.ajhg.2022.10.013 PMC9748256

[18] Brenner SE. Common sense for our genomes. Nature. 2007;449(7164):783–4. 17943102 10.1038/449783a

[19] Hu J, Ng PC. SIFT Indel: predictions for the functional effects of amino acid insertions/deletions in proteins. PLoS ONE. 2013;8(10): e77940. 24194902 10.1371/journal.pone.0077940 PMC3806772

[20] Kumar P, Henikoff S, Ng PC. Predicting the effects of coding non-synonymous variants on protein function using the SIFT algorithm. Nat Protoc. 2009;4(7):1073–81. 19561590 10.1038/nprot.2009.86

[21] Ng PC, Henikoff S. SIFT: Predicting amino acid changes that affect protein function. Nucl Acids Res. 2003;31(13):3812–4. 12824425 10.1093/nar/gkg509 PMC168916

[22] Sim NL, Kumar P, Hu J, Henikoff S, Schneider G, Ng PC. SIFT web server: predicting effects of amino acid substitutions on proteins. Nucl Acids Res. 2012;40(Web Server issue):W452–7. 22689647 10.1093/nar/gks539 PMC3394338

[23] Vaser R, Adusumalli S, Leng SN, Sikic M, Ng PC. SIFT missense predictions for genomes. Nat Protoc. 2016;11(1):1–9. 26633127 10.1038/nprot.2015.123

[24] Adzhubei I, Jordan DM, Sunyaev SR. Predicting functional effect of human missense mutations using PolyPhen-2. Curr Protoc Hum Genet. 2013;Chapter 7:Unit7 20 10.1002/0471142905.hg0720s76 PMC4480630 23315928

[25] Adzhubei IA, Schmidt S, Peshkin L, Ramensky VE, Gerasimova A, Bork P, et al. A method and server for predicting damaging missense mutations. Nat Methods. 2010;7(4):248–9. 20354512 10.1038/nmeth0410-248 PMC2855889

[26] Ramensky V, Bork P, Sunyaev S. Human non-synonymous SNPs: server and survey. Nucl Acids Res. 2002;30(17):3894–900. 12202775 10.1093/nar/gkf493 PMC137415

[27] Wang K, Li M, Hakonarson H. ANNOVAR: functional annotation of genetic variants from high-throughput sequencing data. Nucl Acids Res. 2010;38(16): e164. 20601685 10.1093/nar/gkq603 PMC2938201

[28] Yang H, Wang K. Genomic variant annotation and prioritization with ANNOVAR and wANNOVAR. Nat Protoc. 2015;10(10):1556–66. 26379229 10.1038/nprot.2015.105 PMC4718734

[29] Cingolani P, Patel VM, Coon M, Nguyen T, Land SJ, Ruden DM, et al. Using drosophila melanogaster as a model for genotoxic chemical mutational studies with a new program. SnpSift Front Genet. 2012;3:35. 22435069 10.3389/fgene.2012.00035 PMC3304048

[30] Cingolani P, Platts A, le Wang L, Coon M, Nguyen T, Wang L, et al. A program for annotating and predicting the effects of single nucleotide polymorphisms, SnpEff: SNPs in the genome of Drosophila melanogaster strain w1118; iso-2; iso-3. Fly (Austin). 2012;6(2):80–92. 22728672 10.4161/fly.19695 PMC3679285

[31] Acharya V, Nagarajaram HA. Hansa: an automated method for discriminating disease and neutral human nsSNPs. Hum Mutat. 2012;33(2):332–7. 22045683 10.1002/humu.21642

[32] Ali H, Urolagin S, Gurarslan O, Vihinen M. Performance of protein disorder prediction programs on amino acid substitutions. Hum Mutat. 2014;35(7):794–804. 24753228 10.1002/humu.22564

[33] Alirezaie N, Kernohan KD, Hartley T, Majewski J, Hocking TD. ClinPred: prediction tool to identify disease-relevant nonsynonymous single-nucleotide variants. Am J Hum Genet. 2018;103(4):474–83. 30220433 10.1016/j.ajhg.2018.08.005 PMC6174354

[34] Balasubramanian S, Fu Y, Pawashe M, McGillivray P, Jin M, Liu J, et al. Using ALoFT to determine the impact of putative loss-of-function variants in protein-coding genes. Nat Commun. 2017;8(1):382. 28851873 10.1038/s41467-017-00443-5 PMC5575292

[35] Bamford S, Dawson E, Forbes S, Clements J, Pettett R, Dogan A, et al. The COSMIC (Catalogue of Somatic Mutations in Cancer) database and website. Br J Cancer. 2004;91(2):355–8. 15188009 10.1038/sj.bjc.6601894 PMC2409828

[36] Bao L, Zhou M, Cui Y. nsSNPAnalyzer: identifying disease-associated nonsynonymous single nucleotide polymorphisms. Nucl Acids Res. 2005;33(Web Server issue):W480–2. 15980516 10.1093/nar/gki372 PMC1160133

[37] Barenboim M, Manke T. ChroMoS: an integrated web tool for SNP classification, prioritization and functional interpretation. Bioinformatics. 2013;29(17):2197–8. 23782616 10.1093/bioinformatics/btt356 PMC3740627

[38] Bendl J, Musil M, Stourac J, Zendulka J, Damborsky J, Brezovsky J. PredictSNP2: a unified platform for accurately evaluating SNP effects by exploiting the different characteristics of variants in distinct genomic regions. PLoS Comput Biol. 2016;12(5): e1004962. 27224906 10.1371/journal.pcbi.1004962 PMC4880439

[39] Bendl J, Stourac J, Salanda O, Pavelka A, Wieben ED, Zendulka J, et al. PredictSNP: robust and accurate consensus classifier for prediction of disease-related mutations. PLoS Comput Biol. 2014;10(1): e1003440. 24453961 10.1371/journal.pcbi.1003440 PMC3894168

[40] Bendtsen JD, Nielsen H, von Heijne G, Brunak S. Improved prediction of signal peptides: SignalP 3.0. J Mol Biol. 2004;340(4):783–95. 15223320 10.1016/j.jmb.2004.05.028

[41] Bermejo-Das-Neves C, Nguyen HN, Poch O, Thompson JD. A comprehensive study of small non-frameshift insertions/deletions in proteins and prediction of their phenotypic effects by a machine learning method (KD4i). BMC Bioinform. 2014;15:111. 10.1186/1471-2105-15-111 PMC4021375 24742296

[42] Bertoldi L, Forcato C, Vitulo N, Birolo G, De Pascale F, Feltrin E, et al. QueryOR: a comprehensive web platform for genetic variant analysis and prioritization. BMC Bioinform. 2017;18(1):225. 10.1186/s12859-017-1654-4 PMC5410040 28454514

[43] Bromberg Y, Rost B. SNAP: predict effect of non-synonymous polymorphisms on function. Nucl Acids Res. 2007;35(11):3823–35. 17526529 10.1093/nar/gkm238 PMC1920242

[44] Buske OJ, Manickaraj A, Mital S, Ray PN, Brudno M. Identification of deleterious synonymous variants in human genomes. Bioinformatics. 2013;29(15):1843–50. 23736532 10.1093/bioinformatics/btt308

[45] Capriotti E, Altman RB. A new disease-specific machine learning approach for the prediction of cancer-causing missense variants. Genomics. 2011;98(4):310–7. 21763417 10.1016/j.ygeno.2011.06.010 PMC3371640

[46] Capriotti E, Calabrese R, Casadio R. Predicting the insurgence of human genetic diseases associated to single point protein mutations with support vector machines and evolutionary information. Bioinformatics. 2006;22(22):2729–34. 16895930 10.1093/bioinformatics/btl423

[47] Capriotti E, Calabrese R, Fariselli P, Martelli PL, Altman RB, Casadio R. WS-SNPs&GO: a web server for predicting the deleterious effect of human protein variants using functional annotation. BMC Genom. 2013;14 Suppl 3(Suppl 3):S6. 10.1186/1471-2164-14-S3-S6 PMC3665478 23819482

[48] Capriotti E, Casadio R. K-Fold: a tool for the prediction of the protein folding kinetic order and rate. Bioinformatics. 2007;23(3):385–6. 17138584 10.1093/bioinformatics/btl610

[49] Capriotti E, Fariselli P, Calabrese R, Casadio R. Predicting protein stability changes from sequences using support vector machines. Bioinformatics. 2005;21(Suppl 2):ii54–8. 16204125 10.1093/bioinformatics/bti1109

[50] Cariaso M, Lennon G. SNPedia: a wiki supporting personal genome annotation, interpretation and analysis. Nucl Acids Res. 2012;40(Database issue):D1308–12. 22140107 10.1093/nar/gkr798 PMC3245045

[51] Carter H, Chen S, Isik L, Tyekucheva S, Velculescu VE, Kinzler KW, et al. Cancer-specific high-throughput annotation of somatic mutations: computational prediction of driver missense mutations. Cancer Res. 2009;69(16):6660–7. 19654296 10.1158/0008-5472.CAN-09-1133 PMC2763410

[52] Carter H, Douville C, Stenson PD, Cooper DN, Karchin R. Identifying Mendelian disease genes with the variant effect scoring tool. BMC Genomics. 2013;14 Suppl 3(Suppl 3):S3. 23819870 10.1186/1471-2164-14-S3-S3 PMC3665549

[53] Chelala C, Khan A, Lemoine NR. SNPnexus: a web database for functional annotation of newly discovered and public domain single nucleotide polymorphisms. Bioinformatics. 2009;25(5):655–61. 19098027 10.1093/bioinformatics/btn653 PMC2647830

[54] Cheng J, Nguyen TYD, Cygan KJ, Celik MH, Fairbrother WG, Avsec Z, et al. MMSplice: modular modeling improves the predictions of genetic variant effects on splicing. Genome Biol. 2019;20(1):48. 30823901 10.1186/s13059-019-1653-z PMC6396468

[55] Cheng J, Randall A, Baldi P. Prediction of protein stability changes for single-site mutations using support vector machines. Proteins. 2006;62(4):1125–32. 16372356 10.1002/prot.20810

[56] Choi Y, Chan AP. PROVEAN web server: a tool to predict the functional effect of amino acid substitutions and indels. Bioinformatics. 2015;31(16):2745–7. 25851949 10.1093/bioinformatics/btv195 PMC4528627

[57] Choi Y, Sims GE, Murphy S, Miller JR, Chan AP. Predicting the functional effect of amino acid substitutions and indels. PLoS ONE. 2012;7(10): e46688. 23056405 10.1371/journal.pone.0046688 PMC3466303

[58] Conchillo-Sole O, de Groot NS, Aviles FX, Vendrell J, Daura X, Ventura S. AGGRESCAN: a server for the prediction and evaluation of “hot spots” of aggregation in polypeptides. BMC Bioinform. 2007;8:65. 10.1186/1471-2105-8-65 PMC1828741 17324296

[59] Cuff AL, Janes RW, Martin AC. Analysing the ability to retain sidechain hydrogen-bonds in mutant proteins. Bioinformatics. 2006;22(12):1464–70. 16601005 10.1093/bioinformatics/btl120

[60] Davydov EV, Goode DL, Sirota M, Cooper GM, Sidow A, Batzoglou S. Identifying a high fraction of the human genome to be under selective constraint using GERP++. PLoS Comput Biol. 2010;6(12): e1001025. 21152010 10.1371/journal.pcbi.1001025 PMC2996323

[61] Dayem Ullah AZ, Lemoine NR, Chelala C. SNPnexus: a web server for functional annotation of novel and publicly known genetic variants (2012 update). Nucl Acids Res. 2012;40(Web Server issue):W65-70. 22544707 10.1093/nar/gks364 PMC3394262

[62] Dayem Ullah AZ, Lemoine NR, Chelala C. A practical guide for the functional annotation of genetic variations using SNPnexus. Brief Bioinform. 2013;14(4):437–47. 23395730 10.1093/bib/bbt004

[63] Dayem Ullah AZ, Oscanoa J, Wang J, Nagano A, Lemoine NR, Chelala C. SNPnexus: assessing the functional relevance of genetic variation to facilitate the promise of precision medicine. Nucl Acids Res. 2018;46(W1):W109–13. 29757393 10.1093/nar/gky399 PMC6030955

[64] De Baets G, Van Durme J, Reumers J, Maurer-Stroh S, Vanhee P, Dopazo J, et al. SNPeffect 4.0: on-line prediction of molecular and structural effects of protein-coding variants. Nucl Acids Res. 2012;40(1):D935–9. 22075996 10.1093/nar/gkr996 PMC3245173

[65] Dees ND, Zhang Q, Kandoth C, Wendl MC, Schierding W, Koboldt DC, et al. MuSiC: identifying mutational significance in cancer genomes. Genome Res. 2012;22(8):1589–98. 22759861 10.1101/gr.134635.111 PMC3409272

[66] Dehouck Y, Kwasigroch JM, Gilis D, Rooman M. PoPMuSiC 2.1: a web server for the estimation of protein stability changes upon mutation and sequence optimality. BMC Bioinform. 2011;12:151. 10.1186/1471-2105-12-151 PMC3113940 21569468

[67] Dehouck Y, Kwasigroch JM, Rooman M, Gilis D. BeAtMuSiC: prediction of changes in protein-protein binding affinity on mutations. Nucl Acids Res. 2013;41(Web Server issue):W333–9. 23723246 10.1093/nar/gkt450 PMC3692068

[68] Desmet FO, Hamroun D, Lalande M, Collod-Beroud G, Claustres M, Beroud C. Human Splicing Finder: an online bioinformatics tool to predict splicing signals. Nucl Acids Res. 2009;37(9): e67. 19339519 10.1093/nar/gkp215 PMC2685110

[69] Deutsch C, Krishnamoorthy B. Four-body scoring function for mutagenesis. Bioinformatics. 2007;23(22):3009–15. 17921497 10.1093/bioinformatics/btm481

[70] Dharanipragada P, Seelam SR, Parekh N. SeqVItA: sequence variant identification and annotation platform for next generation sequencing data. Front Genet. 2018;9:537. 30487811 10.3389/fgene.2018.00537 PMC6247818

[71] Dong C, Wei P, Jian X, Gibbs R, Boerwinkle E, Wang K, et al. Comparison and integration of deleteriousness prediction methods for nonsynonymous SNVs in whole exome sequencing studies. Hum Mol Genet. 2015;24(8):2125–37. 25552646 10.1093/hmg/ddu733 PMC4375422

[72] Dosztanyi Z, Magyar C, Tusnady G, Simon I. SCide: identification of stabilization centers in proteins. Bioinformatics. 2003;19(7):899–900. 12724305 10.1093/bioinformatics/btg110

[73] Douville C, Masica DL, Stenson PD, Cooper DN, Gygax DM, Kim R, et al. Assessing the pathogenicity of insertion and deletion variants with the variant effect scoring tool (VEST-Indel). Hum Mutat. 2016;37(1):28–35. 26442818 10.1002/humu.22911 PMC5057310

[74] Dunlavy DM, O’Leary DP, Klimov D, Thirumalai D. HOPE: a homotopy optimization method for protein structure prediction. J Comput Biol. 2005;12(10):1275–88. 16379534 10.1089/cmb.2005.12.1275

[75] Emanuelsson O, Brunak S, von Heijne G, Nielsen H. Locating proteins in the cell using TargetP, SignalP and related tools. Nat Protoc. 2007;2(4):953–71. 17446895 10.1038/nprot.2007.131

[76] Fang Y, Gao S, Tai D, Middaugh CR, Fang J. Identification of properties important to protein aggregation using feature selection. BMC Bioinformatics. 2013;14:314. 24165390 10.1186/1471-2105-14-314 PMC3819749

[77] Fariselli P, Martelli PL, Savojardo C, Casadio R. INPS: predicting the impact of non-synonymous variations on protein stability from sequence. Bioinformatics. 2015;31(17):2816–21. 25957347 10.1093/bioinformatics/btv291

[78] Fernandez-Escamilla AM, Rousseau F, Schymkowitz J, Serrano L. Prediction of sequence-dependent and mutational effects on the aggregation of peptides and proteins. Nat Biotechnol. 2004;22(10):1302–6. 15361882 10.1038/nbt1012

[79] Ferrer-Costa C, Gelpi JL, Zamakola L, Parraga I, de la Cruz X, Orozco M. PMUT: a web-based tool for the annotation of pathological mutations on proteins. Bioinformatics. 2005;21(14):3176–8. 15879453 10.1093/bioinformatics/bti486

[80] Fokkema IF, den Dunnen JT, Taschner PE. LOVD: easy creation of a locus-specific sequence variation database using an “LSDB-in-a-box” approach. Hum Mutat. 2005;26(2):63–8. 15977173 10.1002/humu.20201

[81] Fokkema IF, Taschner PE, Schaafsma GC, Celli J, Laros JF, den Dunnen JT. LOVD v.2.0: the next generation in gene variant databases. Hum Mutat. 2011;32(5):557–63. 21520333 10.1002/humu.21438

[82] Folkman L, Yang Y, Li Z, Stantic B, Sattar A, Mort M, et al. DDIG-in: detecting disease-causing genetic variations due to frameshifting indels and nonsense mutations employing sequence and structural properties at nucleotide and protein levels. Bioinformatics. 2015;31(10):1599–606. 25573915 10.1093/bioinformatics/btu862

[83] Forbes SA, Bhamra G, Bamford S, Dawson E, Kok C, Clements J, et al. The Catalogue of Somatic Mutations in Cancer (COSMIC). Curr Protoc Hum Genet. 2008;Chapter 10:Unit 10 1. 10.1002/0471142905.hg1011s57 PMC2705836 18428421

[84] Frederic MY, Lalande M, Boileau C, Hamroun D, Claustres M, Beroud C, et al. UMD-predictor, a new prediction tool for nucleotide substitution pathogenicity – application to four genes: FBN1, FBN2, TGFBR1, and TGFBR2. Hum Mutat. 2009;30(6):952–9. 19370756 10.1002/humu.20970

[85] Frousios K, Iliopoulos CS, Schlitt T, Simpson MA. Predicting the functional consequences of non-synonymous DNA sequence variants–evaluation of bioinformatics tools and development of a consensus strategy. Genomics. 2013;102(4):223–8. 23831115 10.1016/j.ygeno.2013.06.005

[86] Gao M, Skolnick J. DBD-Hunter: a knowledge-based method for the prediction of DNA-protein interactions. Nucl Acids Res. 2008;36(12):3978–92. 18515839 10.1093/nar/gkn332 PMC2475642

[87] Garber M, Guttman M, Clamp M, Zody MC, Friedman N, Xie X. Identifying novel constrained elements by exploiting biased substitution patterns. Bioinformatics. 2009;25(12):i54-62. 19478016 10.1093/bioinformatics/btp190 PMC2687944

[88] Garbuzynskiy SO, Lobanov MY, Galzitskaya OV. FoldAmyloid: a method of prediction of amyloidogenic regions from protein sequence. Bioinformatics. 2010;26(3):326–32. 20019059 10.1093/bioinformatics/btp691

[89] Genomes Project C, Abecasis GR, Altshuler D, Auton A, Brooks LD, Durbin RM, et al. A map of human genome variation from population-scale sequencing. Nature. 2010;467(7319):1061–73. 20981092 10.1038/nature09534 PMC3042601

[90] Giollo M, Martin AJ, Walsh I, Ferrari C, Tosatto SC. NeEMO: a method using residue interaction networks to improve prediction of protein stability upon mutation. BMC Genomics. 2014;15 Suppl 4(Suppl 4):S7. 25057121 10.1186/1471-2164-15-S4-S7 PMC4083412

[91] Goldberg T, Hamp T, Rost B. LocTree2 predicts localization for all domains of life. Bioinformatics. 2012;28(18):i458–65. 22962467 10.1093/bioinformatics/bts390 PMC3436817

[92] Goldberg T, Hecht M, Hamp T, Karl T, Yachdav G, Ahmed N, et al. LocTree3 prediction of localization. Nucl Acids Res. 2014;42(Web Server issue):W350–5. 24848019 10.1093/nar/gku396 PMC4086075

[93] Gonzalez-Perez A, Deu-Pons J, Lopez-Bigas N. Improving the prediction of the functional impact of cancer mutations by baseline tolerance transformation. Genome Med. 2012;4(11):89. 23181723 10.1186/gm390 PMC4064314

[94] Gonzalez-Perez A, Lopez-Bigas N. Improving the assessment of the outcome of nonsynonymous SNVs with a consensus deleteriousness score. Condel Am J Hum Genet. 2011;88(4):440–9. 21457909 10.1016/j.ajhg.2011.03.004 PMC3071923

[95] Gosalia N, Economides AN, Dewey FE, Balasubramanian S. MAPPIN: a method for annotating, predicting pathogenicity and mode of inheritance for nonsynonymous variants. Nucl Acids Res. 2017;45(18):10393–402. 28977528 10.1093/nar/gkx730 PMC5737764

[96] Gromiha MM, Thangakani AM, Selvaraj S. FOLD-RATE: prediction of protein folding rates from amino acid sequence. Nucl Acids Res. 2006;34(Web Server issue):W70–4. 16845101 10.1093/nar/gkl043 PMC1538837

[97] Gulko B, Hubisz MJ, Gronau I, Siepel A. A method for calculating probabilities of fitness consequences for point mutations across the human genome. Nat Genet. 2015;47(3):276–83. 25599402 10.1038/ng.3196 PMC4342276

[98] Hamosh A, Scott AF, Amberger J, Valle D, McKusick VA. Online mendelian inheritance in man (OMIM). Hum Mutat. 2000;15(1):57–61. 10612823 10.1002/(SICI)1098-1004(200001)15:1<57::AID-HUMU12>3.0.CO;2-G

[99] Hecht M, Bromberg Y, Rost B. News from the protein mutability landscape. J Mol Biol. 2013;425(21):3937–48. 23896297 10.1016/j.jmb.2013.07.028

[100] Hecht M, Bromberg Y, Rost B. Better prediction of functional effects for sequence variants. BMC Genomics. 2015;16 Suppl 8(Suppl 8):S1. 26110438 10.1186/1471-2164-16-S8-S1 PMC4480835

[101] Hopf TA, Ingraham JB, Poelwijk FJ, Scharfe CP, Springer M, Sander C, et al. Mutation effects predicted from sequence co-variation. Nat Biotechnol. 2017;35(2):128–35. 28092658 10.1038/nbt.3769 PMC5383098

[102] Horton P, Park KJ, Obayashi T, Fujita N, Harada H, Adams-Collier CJ, et al. WoLF PSORT: protein localization predictor. Nucl Acids Res. 2007;35(Web Server issue):W585–7. 17517783 10.1093/nar/gkm259 PMC1933216

[103] Hu H, Huff CD, Moore B, Flygare S, Reese MG, Yandell M. VAAST 2.0: improved variant classification and disease-gene identification using a conservation-controlled amino acid substitution matrix. Genet Epidemiol. 2013;37(6):622–34. 23836555 10.1002/gepi.21743 PMC3791556

[104] Hurst JM, McMillan LE, Porter CT, Allen J, Fakorede A, Martin AC. The SAAPdb web resource: a large-scale structural analysis of mutant proteins. Hum Mutat. 2009;30(4):616–24. 19191322 10.1002/humu.20898

[105] Ioannidis NM, Rothstein JH, Pejaver V, Middha S, McDonnell SK, Baheti S, et al. REVEL: an ensemble method for predicting the pathogenicity of rare missense variants. Am J Hum Genet. 2016;99(4):877–85. 27666373 10.1016/j.ajhg.2016.08.016 PMC5065685

[106] Ionita-Laza I, McCallum K, Xu B, Buxbaum JD. A spectral approach integrating functional genomic annotations for coding and noncoding variants. Nat Genet. 2016;48(2):214–20. 26727659 10.1038/ng.3477 PMC4731313

[107] Javed A, Agrawal S, Ng PC. Phen-Gen: combining phenotype and genotype to analyze rare disorders. Nat Methods. 2014;11(9):935–7. 25086502 10.1038/nmeth.3046

[108] Jia P, Zhao Z. VarWalker: personalized mutation network analysis of putative cancer genes from next-generation sequencing data. PLoS Comput Biol. 2014;10(2): e1003460. 24516372 10.1371/journal.pcbi.1003460 PMC3916227

[109] Jian X, Boerwinkle E, Liu X. In silico prediction of splice-altering single nucleotide variants in the human genome. Nucl Acids Res. 2014;42(22):13534–44. 25416802 10.1093/nar/gku1206 PMC4267638

[110] Johansen MB, Izarzugaza JM, Brunak S, Petersen TN, Gupta R. Prediction of disease causing non-synonymous SNPs by the Artificial Neural Network Predictor NetDiseaseSNP. PLoS ONE. 2013;8(7): e68370. 23935863 10.1371/journal.pone.0068370 PMC3723835

[111] Kaminker JS, Zhang Y, Watanabe C, Zhang Z. CanPredict: a computational tool for predicting cancer-associated missense mutations. Nucl Acids Res. 2007;35(Web Server issue):W595–8. 17537827 10.1093/nar/gkm405 PMC1933186

[112] Kang S, Chen G, Xiao G. Robust prediction of mutation-induced protein stability change by property encoding of amino acids. Protein Eng Des Sel. 2009;22(2):75–83. 19054789 10.1093/protein/gzn063

[113] Karczewski KJ, Francioli LC, Tiao G, Cummings BB, Alfoldi J, Wang Q, et al. The mutational constraint spectrum quantified from variation in 141,456 humans. Nature. 2020;581(7809):434–43. 32461654 10.1038/s41586-020-2308-7 PMC7334197

[114] Kircher M, Witten DM, Jain P, O’Roak BJ, Cooper GM, Shendure J. A general framework for estimating the relative pathogenicity of human genetic variants. Nat Genet. 2014;46(3):310–5. 24487276 10.1038/ng.2892 PMC3992975

[115] Knecht C, Mort M, Junge O, Cooper DN, Krawczak M, Caliebe A. IMHOTEP-a composite score integrating popular tools for predicting the functional consequences of non-synonymous sequence variants. Nucl Acids Res. 2017;45(3): e13. 28180317 10.1093/nar/gkw886 PMC5388428

[116] Krassowski M, Paczkowska M, Cullion K, Huang T, Dzneladze I, Ouellette BFF, et al. ActiveDriverDB: human disease mutations and genome variation in post-translational modification sites of proteins. Nucl Acids Res. 2018;46(D1):D901–10. 29126202 10.1093/nar/gkx973 PMC5753267

[117] Kulandaisamy A, Zaucha J, Sakthivel R, Frishman D, Michael GM. Pred-MutHTP: prediction of disease-causing and neutral mutations in human transmembrane proteins. Hum Mutat. 2020;41(3):581–90. 31821684 10.1002/humu.23961

[118] Kurgan L, Cios K, Chen K. SCPRED: accurate prediction of protein structural class for sequences of twilight-zone similarity with predicting sequences. BMC Bioinform. 2008;9:226. 10.1186/1471-2105-9-226 PMC2391167 18452616

[119] Laimer J, Hofer H, Fritz M, Wegenkittl S, Lackner P. MAESTRO–multi agent stability prediction upon point mutations. BMC Bioinform. 2015;16:116. 10.1186/s12859-015-0548-6 PMC4403899 25885774

[120] Landrum MJ, Lee JM, Benson M, Brown G, Chao C, Chitipiralla S, et al. ClinVar: public archive of interpretations of clinically relevant variants. Nucl Acids Res. 2016;44(D1):D862–8. 26582918 10.1093/nar/gkv1222 PMC4702865

[121] Lappalainen I, Lopez J, Skipper L, Hefferon T, Spalding JD, Garner J, et al. DbVar and DGVa: public archives for genomic structural variation. Nucl Acids Res. 2013;41(Database issue):D936–41. 23193291 10.1093/nar/gks1213 PMC3531204

[122] Lawrence MS, Stojanov P, Polak P, Kryukov GV, Cibulskis K, Sivachenko A, et al. Mutational heterogeneity in cancer and the search for new cancer-associated genes. Nature. 2013;499(7457):214–8. 23770567 10.1038/nature12213 PMC3919509

[123] Lehmann KV, Chen T. Exploring functional variant discovery in non-coding regions with SInBaD. Nucl Acids Res. 2013;41(1): e7. 22941663 10.1093/nar/gks800 PMC3592431

[124] Leiserson MD, Wu HT, Vandin F, Raphael BJ. CoMEt: a statistical approach to identify combinations of mutually exclusive alterations in cancer. Genome Biol. 2015;16(1):160. 26253137 10.1186/s13059-015-0700-7 PMC4531541

[125] Li B, Krishnan VG, Mort ME, Xin F, Kamati KK, Cooper DN, et al. Automated inference of molecular mechanisms of disease from amino acid substitutions. Bioinformatics. 2009;25(21):2744–50. 19734154 10.1093/bioinformatics/btp528 PMC3140805

[126] Li MJ, Li M, Liu Z, Yan B, Pan Z, Huang D, et al. cepip: context-dependent epigenomic weighting for prioritization of regulatory variants and disease-associated genes. Genome Biol. 2017;18(1):52. 28302177 10.1186/s13059-017-1177-3 PMC5356314

[127] Li MJ, Pan Z, Liu Z, Wu J, Wang P, Zhu Y, et al. Predicting regulatory variants with composite statistic. Bioinformatics. 2016;32(18):2729–36. 27273672 10.1093/bioinformatics/btw288 PMC6280872

[128] Li MX, Kwan JS, Bao SY, Yang W, Ho SL, Song YQ, et al. Predicting mendelian disease-causing non-synonymous single nucleotide variants in exome sequencing studies. PLoS Genet. 2013;9(1): e1003143. 23341771 10.1371/journal.pgen.1003143 PMC3547823

[129] Li Q, Wang K. InterVar: clinical interpretation of genetic variants by the 2015 ACMG-AMP guidelines. Am J Hum Genet. 2017;100(2):267–80. 28132688 10.1016/j.ajhg.2017.01.004 PMC5294755

[130] Linding R, Schymkowitz J, Rousseau F, Diella F, Serrano L. A comparative study of the relationship between protein structure and beta-aggregation in globular and intrinsically disordered proteins. J Mol Biol. 2004;342(1):345–53. 15313629 10.1016/j.jmb.2004.06.088

[131] Liu M, Watson LT, Zhang L. Predicting the combined effect of multiple genetic variants. Hum Genomics. 2015;9(1):18. 26223264 10.1186/s40246-015-0040-4 PMC4520001

[132] Liu X, Jian X, Boerwinkle E. dbNSFP: a lightweight database of human nonsynonymous SNPs and their functional predictions. Hum Mutat. 2011;32(8):894–9. 21520341 10.1002/humu.21517 PMC3145015

[133] Liu X, Jian X, Boerwinkle E. dbNSFP v2.0: a database of human non-synonymous SNVs and their functional predictions and annotations. Hum Mutat. 2013;34(9):E2393–402. 23843252 10.1002/humu.22376 PMC4109890

[134] Liu X, White S, Peng B, Johnson AD, Brody JA, Li AH, et al. WGSA: an annotation pipeline for human genome sequencing studies. J Med Genet. 2016;53(2):111–2. 26395054 10.1136/jmedgenet-2015-103423 PMC5124490

[135] Liu X, Wu C, Li C, Boerwinkle E. dbNSFP v3.0: a one-stop database of functional predictions and annotations for human nonsynonymous and splice-site SNVs. Hum Mutat. 2016;37(3):235–41. 26555599 10.1002/humu.22932 PMC4752381

[136] Livingstone M, Folkman L, Yang Y, Zhang P, Mort M, Cooper DN, et al. Investigating DNA-, RNA-, and protein-based features as a means to discriminate pathogenic synonymous variants. Hum Mutat. 2017;38(10):1336–47. 28649752 10.1002/humu.23283

[137] Lopes MC, Joyce C, Ritchie GR, John SL, Cunningham F, Asimit J, et al. A combined functional annotation score for non-synonymous variants. Hum Hered. 2012;73(1):47–51. 22261837 10.1159/000334984 PMC3390741

[138] Lopez-Ferrando V, Gazzo A, de la Cruz X, Orozco M, Gelpi JL. PMut: a web-based tool for the annotation of pathological variants on proteins, 2017 update. Nucl Acids Res. 2017;45(W1):W222–8. 28453649 10.1093/nar/gkx313 PMC5793831

[139] Lu Q, Hu Y, Sun J, Cheng Y, Cheung KH, Zhao H. A statistical framework to predict functional non-coding regions in the human genome through integrated analysis of annotation data. Sci Rep. 2015;5:10576. 26015273 10.1038/srep10576 PMC4444969

[140] Macintyre G, Bailey J, Haviv I, Kowalczyk A. is-rSNP: a novel technique for in silico regulatory SNP detection. Bioinformatics. 2010;26(18):i524–30. 20823317 10.1093/bioinformatics/btq378 PMC2935445

[141] Mao Y, Chen H, Liang H, Meric-Bernstam F, Mills GB, Chen K. CanDrA: cancer-specific driver missense mutation annotation with optimized features. PLoS ONE. 2013;8(10): e77945. 24205039 10.1371/journal.pone.0077945 PMC3813554

[142] Marini NJ, Thomas PD, Rine J. The use of orthologous sequences to predict the impact of amino acid substitutions on protein function. PLoS Genet. 2010;6(5): e1000968. 20523748 10.1371/journal.pgen.1000968 PMC2877731

[143] Masso M, Vaisman II. AUTO-MUTE: web-based tools for predicting stability changes in proteins due to single amino acid replacements. Protein Eng Des Sel. 2010;23(8):683–7. 20573719 10.1093/protein/gzq042

[144] Mathe E, Olivier M, Kato S, Ishioka C, Hainaut P, Tavtigian SV. Computational approaches for predicting the biological effect of p53 missense mutations: a comparison of three sequence analysis based methods. Nucl Acids Res. 2006;34(5):1317–25. 16522644 10.1093/nar/gkj518 PMC1390679

[145] Maurer-Stroh S, Debulpaep M, Kuemmerer N, Lopez de la Paz M, Martins IC, Reumers J, et al. Exploring the sequence determinants of amyloid structure using position-specific scoring matrices. Nat Methods. 2010;7(3):237–42. 20154676 10.1038/nmeth.1432

[146] McLaren W, Gil L, Hunt SE, Riat HS, Ritchie GR, Thormann A, et al. The Ensembl Variant Effect Predictor. Genome Biol. 2016;17(1):122. 27268795 10.1186/s13059-016-0974-4 PMC4893825

[147] McLaren W, Pritchard B, Rios D, Chen Y, Flicek P, Cunningham F. Deriving the consequences of genomic variants with the Ensembl API and SNP Effect Predictor. Bioinformatics. 2010;26(16):2069–70. 20562413 10.1093/bioinformatics/btq330 PMC2916720

[148] Mi H, Huang X, Muruganujan A, Tang H, Mills C, Kang D, et al. PANTHER version 11: expanded annotation data from Gene Ontology and Reactome pathways, and data analysis tool enhancements. Nucl Acids Res. 2017;45(D1):D183–9. 27899595 10.1093/nar/gkw1138 PMC5210595

[149] Moretti R, Fleishman SJ, Agius R, Torchala M, Bates PA, Kastritis PL, et al. Community-wide evaluation of methods for predicting the effect of mutations on protein-protein interactions. Proteins. 2013;81(11):1980–7. 23843247 10.1002/prot.24356 PMC4143140

[150] Mort M, Sterne-Weiler T, Li B, Ball EV, Cooper DN, Radivojac P, et al. MutPred Splice: machine learning-based prediction of exonic variants that disrupt splicing. Genome Biol. 2014;15(1):R19. 24451234 10.1186/gb-2014-15-1-r19 PMC4054890

[151] Nalla VK, Rogan PK. Automated splicing mutation analysis by information theory. Hum Mutat. 2005;25(4):334–42. 15776446 10.1002/humu.20151

[152] Nielsen H, Krogh A. Prediction of signal peptides and signal anchors by a hidden Markov model. Proc Int Conf Intell Syst Mol Biol. 1998;6:122–30. 9783217

[153] Niroula A, Urolagin S, Vihinen M. PON-P2: prediction method for fast and reliable identification of harmful variants. PLoS ONE. 2015;10(2): e0117380. 25647319 10.1371/journal.pone.0117380 PMC4315405

[154] Niroula A, Vihinen M. PON-mt-tRNA: a multifactorial probability-based method for classification of mitochondrial tRNA variations. Nucl Acids Res. 2016;44(5):2020–7. 26843426 10.1093/nar/gkw046 PMC4797295

[155] Olatubosun A, Valiaho J, Harkonen J, Thusberg J, Vihinen M. PON-P: integrated predictor for pathogenicity of missense variants. Hum Mutat. 2012;33(8):1166–74. 22505138 10.1002/humu.22102

[156] Pagel KA, Pejaver V, Lin GN, Nam HJ, Mort M, Cooper DN, et al. When loss-of-function is loss of function: assessing mutational signatures and impact of loss-of-function genetic variants. Bioinformatics. 2017;33(14):i389–98. 28882004 10.1093/bioinformatics/btx272 PMC5870554

[157] Pagon RA, Tarczy-Hornoch P, Baskin PK, Edwards JE, Covington ML, Espeseth M, et al. GeneTests-GeneClinics: genetic testing information for a growing audience. Hum Mutat. 2002;19(5):501–9. 11968082 10.1002/humu.10069

[158] Pandurangan AP, Ochoa-Montano B, Ascher DB, Blundell TL. SDM: a server for predicting effects of mutations on protein stability. Nucl Acids Res. 2017;45(W1):W229–35. 28525590 10.1093/nar/gkx439 PMC5793720

[159] Pappalardo M, Wass MN. VarMod: modelling the functional effects of non-synonymous variants. Nucl Acids Res. 2014;42(Web Server issue):W331–6. 24906884 10.1093/nar/gku483 PMC4086131

[160] Parthiban V, Gromiha MM, Abhinandan M, Schomburg D. Computational modeling of protein mutant stability: analysis and optimization of statistical potentials and structural features reveal insights into prediction model development. BMC Struct Biol. 2007;7:54. 17705837 10.1186/1472-6807-7-54 PMC2000882

[161] Parthiban V, Gromiha MM, Hoppe C, Schomburg D. Structural analysis and prediction of protein mutant stability using distance and torsion potentials: role of secondary structure and solvent accessibility. Proteins. 2007;66(1):41–52. 17068801 10.1002/prot.21115

[162] Parthiban V, Gromiha MM, Schomburg D. CUPSAT: prediction of protein stability upon point mutations. Nucl Acids Res. 2006;34(Web Server issue):W239–42. 16845001 10.1093/nar/gkl190 PMC1538884

[163] Pejaver V, Urresti J, Lugo-Martinez J, Pagel KA, Lin GN, Nam HJ, et al. Inferring the molecular and phenotypic impact of amino acid variants with MutPred2. Nat Commun. 2020;11(1):5918. 33219223 10.1038/s41467-020-19669-x PMC7680112

[164] Peng B. Reproducible simulations of realistic samples for next-generation sequencing studies using Variant Simulation Tools. Genet Epidemiol. 2015;39(1):45–52. 25395236 10.1002/gepi.21867 PMC6432799

[165] Petersen TN, Brunak S, von Heijne G, Nielsen H. SignalP 4.0: discriminating signal peptides from transmembrane regions. Nat Methods. 2011;8(10):785–6. 21959131 10.1038/nmeth.1701

[166] Pires DE, Ascher DB, Blundell TL. DUET: a server for predicting effects of mutations on protein stability using an integrated computational approach. Nucl Acids Res. 2014;42(Web Server issue):W314–9. 24829462 10.1093/nar/gku411 PMC4086143

[167] Pokala N, Handel TM. Energy functions for protein design: adjustment with protein-protein complex affinities, models for the unfolded state, and negative design of solubility and specificity. J Mol Biol. 2005;347(1):203–27. 15733929 10.1016/j.jmb.2004.12.019

[168] Pollard KS, Hubisz MJ, Rosenbloom KR, Siepel A. Detection of nonneutral substitution rates on mammalian phylogenies. Genome Res. 2010;20(1):110–21. 19858363 10.1101/gr.097857.109 PMC2798823

[169] Preeprem T, Gibson G. SDS, a structural disruption score for assessment of missense variant deleteriousness. Front Genet. 2014;5:82. 24795746 10.3389/fgene.2014.00082 PMC4001065

[170] Punta M, Rost B. PROFcon: novel prediction of long-range contacts. Bioinformatics. 2005;21(13):2960–8. 15890748 10.1093/bioinformatics/bti454

[171] Qin S, Pang X, Zhou HX. Automated prediction of protein association rate constants. Structure. 2011;19(12):1744–51. 22153497 10.1016/j.str.2011.10.015 PMC3240845

[172] Quang D, Chen Y, Xie X. DANN: a deep learning approach for annotating the pathogenicity of genetic variants. Bioinformatics. 2015;31(5):761–3. 25338716 10.1093/bioinformatics/btu703 PMC4341060

[173] Reumers J, Conde L, Medina I, Maurer-Stroh S, Van Durme J, Dopazo J, et al. Joint annotation of coding and non-coding single nucleotide polymorphisms and mutations in the SNPeffect and PupaSuite databases. Nucl Acids Res. 2008;36(Database issue):D825–9. 18086700 10.1093/nar/gkm979 PMC2238831

[174] Reumers J, Maurer-Stroh S, Schymkowitz J, Rousseau F. SNPeffect v2.0: a new step in investigating the molecular phenotypic effects of human non-synonymous SNPs. Bioinformatics. 2006;22(17):2183–5. 16809394 10.1093/bioinformatics/btl348

[175] Reumers J, Schymkowitz J, Ferkinghoff-Borg J, Stricher F, Serrano L, Rousseau F. SNPeffect: a database mapping molecular phenotypic effects of human non-synonymous coding SNPs. Nucl Acids Res. 2005;33(Database issue):D527–32. 15608254 10.1093/nar/gki086 PMC540040

[176] Reva B, Antipin Y, Sander C. Determinants of protein function revealed by combinatorial entropy optimization. Genome Biol. 2007;8(11):R232. 17976239 10.1186/gb-2007-8-11-r232 PMC2258190

[177] Reva B, Antipin Y, Sander C. Predicting the functional impact of protein mutations: application to cancer genomics. Nucl Acids Res. 2011;39(17): e118. 21727090 10.1093/nar/gkr407 PMC3177186

[178] Ritchie GR, Dunham I, Zeggini E, Flicek P. Functional annotation of noncoding sequence variants. Nat Methods. 2014;11(3):294–6. 24487584 10.1038/nmeth.2832 PMC5015703

[179] Rousseau F, Schymkowitz J, Serrano L. Protein aggregation and amyloidosis: confusion of the kinds? Curr Opin Struct Biol. 2006;16(1):118–26. 16434184 10.1016/j.sbi.2006.01.011

[180] Ryan M, Diekhans M, Lien S, Liu Y, Karchin R. LS-SNP/PDB: annotated non-synonymous SNPs mapped to Protein Data Bank structures. Bioinformatics. 2009;25(11):1431–2. 19369493 10.1093/bioinformatics/btp242 PMC6276889

[181] Ryan NM, Morris SW, Porteous DJ, Taylor MS, Evans KL. SuRFing the genomics wave: an R package for prioritising SNPs by functionality. Genome Med. 2014;6(10):79. 25400697 10.1186/s13073-014-0079-1 PMC4224693

[182] San Lucas FA, Wang G, Scheet P, Peng B. Integrated annotation and analysis of genetic variants from next-generation sequencing studies with variant tools. Bioinformatics. 2012;28(3):421–2. 22138362 10.1093/bioinformatics/btr667 PMC3268240

[183] Sasidharan Nair P, Vihinen M. VariBench: a benchmark database for variations. Hum Mutat. 2013;34(1):42–9. 22903802 10.1002/humu.22204

[184] Savojardo C, Fariselli P, Martelli PL, Casadio R. INPS-MD: a web server to predict stability of protein variants from sequence and structure. Bioinformatics. 2016;32(16):2542–4. 27153629 10.1093/bioinformatics/btw192

[185] Schaafsma GC, Vihinen M. VariSNP, a benchmark database for variations from dbSNP. Hum Mutat. 2015;36(2):161–6. 25385275 10.1002/humu.22727

[186] Schaefer C, Meier A, Rost B, Bromberg Y. SNPdbe: constructing an nsSNP functional impacts database. Bioinformatics. 2012;28(4):601–2. 22210871 10.1093/bioinformatics/btr705 PMC3278761

[187] Schwarz JM, Cooper DN, Schuelke M, Seelow D. MutationTaster2: mutation prediction for the deep-sequencing age. Nat Methods. 2014;11(4):361–2. 24681721 10.1038/nmeth.2890

[188] Schwarz JM, Rodelsperger C, Schuelke M, Seelow D. MutationTaster evaluates disease-causing potential of sequence alterations. Nat Methods. 2010;7(8):575–6. 20676075 10.1038/nmeth0810-575

[189] Schymkowitz J, Borg J, Stricher F, Nys R, Rousseau F, Serrano L. The FoldX web server: an online force field. Nucl Acids Res. 2005;33(Web Server issue):W382–8. 15980494 10.1093/nar/gki387 PMC1160148

[190] Sherry ST, Ward MH, Kholodov M, Baker J, Phan L, Smigielski EM, et al. dbSNP: the NCBI database of genetic variation. Nucl Acids Res. 2001;29(1):308–11. 11125122 10.1093/nar/29.1.308 PMC29783

[191] Shihab HA, Gough J, Cooper DN, Day IN, Gaunt TR. Predicting the functional consequences of cancer-associated amino acid substitutions. Bioinformatics. 2013;29(12):1504–10. 23620363 10.1093/bioinformatics/btt182 PMC3673218

[192] Shihab HA, Gough J, Cooper DN, Stenson PD, Barker GL, Edwards KJ, et al. Predicting the functional, molecular, and phenotypic consequences of amino acid substitutions using hidden Markov models. Hum Mutat. 2013;34(1):57–65. 23033316 10.1002/humu.22225 PMC3558800

[193] Shihab HA, Gough J, Mort M, Cooper DN, Day IN, Gaunt TR. Ranking non-synonymous single nucleotide polymorphisms based on disease concepts. Hum Genomics. 2014;8(1):11. 24980617 10.1186/1479-7364-8-11 PMC4083756

[194] Shihab HA, Rogers MF, Gough J, Mort M, Cooper DN, Day IN, et al. An integrative approach to predicting the functional effects of non-coding and coding sequence variation. Bioinformatics. 2015;31(10):1536–43. 25583119 10.1093/bioinformatics/btv009 PMC4426838

[195] Shringarpure SS, Bustamante CD. Privacy risks from genomic data-sharing beacons. Am J Hum Genet. 2015;97(5):631–46. 26522470 10.1016/j.ajhg.2015.09.010 PMC4667107

[196] Siepel A, Bejerano G, Pedersen JS, Hinrichs AS, Hou M, Rosenbloom K, et al. Evolutionarily conserved elements in vertebrate, insect, worm, and yeast genomes. Genome Res. 2005;15(8):1034–50. 16024819 10.1101/gr.3715005 PMC1182216

[197] Smedley D, Jacobsen JO, Jager M, Kohler S, Holtgrewe M, Schubach M, et al. Next-generation diagnostics and disease-gene discovery with the Exomiser. Nat Protoc. 2015;10(12):2004–15. 26562621 10.1038/nprot.2015.124 PMC5467691

[198] Smedley D, Schubach M, Jacobsen JOB, Kohler S, Zemojtel T, Spielmann M, et al. A whole-genome analysis framework for effective identification of pathogenic regulatory variants in Mendelian disease. Am J Hum Genet. 2016;99(3):595–606. 27569544 10.1016/j.ajhg.2016.07.005 PMC5011059

[199] Stenson PD, Mort M, Ball EV, Evans K, Hayden M, Heywood S, et al. The Human Gene Mutation Database: towards a comprehensive repository of inherited mutation data for medical research, genetic diagnosis and next-generation sequencing studies. Hum Genet. 2017;136(6):665–77. 28349240 10.1007/s00439-017-1779-6 PMC5429360

[200] Stone EA, Sidow A. Physicochemical constraint violation by missense substitutions mediates impairment of protein function and disease severity. Genome Res. 2005;15(7):978–86. 15965030 10.1101/gr.3804205 PMC1172042

[201] Tamborero D, Gonzalez-Perez A, Lopez-Bigas N. OncodriveCLUST: exploiting the positional clustering of somatic mutations to identify cancer genes. Bioinformatics. 2013;29(18):2238–44. 23884480 10.1093/bioinformatics/btt395

[202] Tang H, Thomas PD. PANTHER-PSEP: predicting disease-causing genetic variants using position-specific evolutionary preservation. Bioinformatics. 2016;32(14):2230–2. 27193693 10.1093/bioinformatics/btw222

[203] Tartaglia GG, Cavalli A, Pellarin R, Caflisch A. Prediction of aggregation rate and aggregation-prone segments in polypeptide sequences. Protein Sci. 2005;14(10):2723–34. 16195556 10.1110/ps.051471205 PMC2253302

[204] Tartaglia GG, Vendruscolo M. The Zyggregator method for predicting protein aggregation propensities. Chem Soc Rev. 2008;37(7):1395–401. 18568165 10.1039/b706784b

[205] Tate JG, Bamford S, Jubb HC, Sondka Z, Beare DM, Bindal N, et al. COSMIC: the catalogue of somatic mutations in cancer. Nucl Acids Res. 2019;47(D1):D941–7. 30371878 10.1093/nar/gky1015 PMC6323903

[206] Tavtigian SV, Deffenbaugh AM, Yin L, Judkins T, Scholl T, Samollow PB, et al. Comprehensive statistical study of 452 BRCA1 missense substitutions with classification of eight recurrent substitutions as neutral. J Med Genet. 2006;43(4):295–305. 16014699 10.1136/jmg.2005.033878 PMC2563222

[207] Teng S, Srivastava AK, Wang L. Sequence feature-based prediction of protein stability changes upon amino acid substitutions. BMC Genomics. 2010;11 Suppl 2(Suppl 2):S5. 21047386 10.1186/1471-2164-11-S2-S5 PMC2975416

[208] Terui H, Akagi K, Kawame H, Yura K. CoDP: predicting the impact of unclassified genetic variants in MSH6 by the combination of different properties of the protein. J Biomed Sci. 2013;20(1):25. 23621914 10.1186/1423-0127-20-25 PMC3651391

[209] Thompson BA, Spurdle AB, Plazzer JP, Greenblatt MS, Akagi K, Al-Mulla F, et al. Application of a 5-tiered scheme for standardized classification of 2,360 unique mismatch repair gene variants in the InSiGHT locus-specific database. Nat Genet. 2014;46(2):107–15. 24362816 10.1038/ng.2854 PMC4294709

[210] Thorn CF, Klein TE, Altman RB. PharmGKB: the Pharmacogenomics Knowledge Base. Methods Mol Biol. 2013;1015:311–20. 23824865 10.1007/978-1-62703-435-7_20 PMC4084821

[211] Tian J, Wu N, Guo X, Guo J, Zhang J, Fan Y. Predicting the phenotypic effects of non-synonymous single nucleotide polymorphisms based on support vector machines. BMC Bioinform. 2007;8:450. 10.1186/1471-2105-8-450 PMC2216041 18005451

[212] Vuong H, Che A, Ravichandran S, Luke BT, Collins JR, Mudunuri US. AVIA v2.0: annotation, visualization and impact analysis of genomic variants and genes. Bioinformatics. 2015;31(16):2748–50. 25861966 10.1093/bioinformatics/btv200 PMC4528632

[213] Walsh I, Seno F, Tosatto SC, Trovato A. PASTA 2.0: an improved server for protein aggregation prediction. Nucl Acids Res. 2014;42(Web Server issue):W301–7. 24848016 10.1093/nar/gku399 PMC4086119

[214] Wang GT, Peng B, Leal SM. Variant association tools for quality control and analysis of large-scale sequence and genotyping array data. Am J Hum Genet. 2014;94(5):770–83. 24791902 10.1016/j.ajhg.2014.04.004 PMC4067555

[215] Wang M, Zhao XM, Takemoto K, Xu H, Li Y, Akutsu T, et al. FunSAV: predicting the functional effect of single amino acid variants using a two-stage random forest model. PLoS ONE. 2012;7(8): e43847. 22937107 10.1371/journal.pone.0043847 PMC3427247

[216] Wishart DS, Arndt D, Berjanskii M, Guo AC, Shi Y, Shrivastava S, et al. PPT-DB: the protein property prediction and testing database. Nucl Acids Res. 2008;36(Database issue):D222–9. 17916570 10.1093/nar/gkm800 PMC2238980

[217] Wong WC, Kim D, Carter H, Diekhans M, Ryan MC, Karchin R. CHASM and SNVBox: toolkit for detecting biologically important single nucleotide mutations in cancer. Bioinformatics. 2011;27(15):2147–8. 21685053 10.1093/bioinformatics/btr357 PMC3137226

[218] Woolfe A, Mullikin JC, Elnitski L. Genomic features defining exonic variants that modulate splicing. Genome Biol. 2010;11(2):R20. 20158892 10.1186/gb-2010-11-2-r20 PMC2872880

[219] Xiong HY, Alipanahi B, Lee LJ, Bretschneider H, Merico D, Yuen RK, et al. RNA splicing. The human splicing code reveals new insights into the genetic determinants of disease. Science. 2015;347(6218):1254806. 25525159 10.1126/science.1254806 PMC4362528

[220] Xu B, Yang Y, Liang H, Zhou Y. An all-atom knowledge-based energy function for protein-DNA threading, docking decoy discrimination, and prediction of transcription-factor binding profiles. Proteins. 2009;76(3):718–30. 19274740 10.1002/prot.22384 PMC2743280

[221] Xu Z, Taylor JA. SNPinfo: integrating GWAS and candidate gene information into functional SNP selection for genetic association studies. Nucl Acids Res. 2009;37(Web Server issue):W600–5. 19417063 10.1093/nar/gkp290 PMC2703930

[222] Yandell M, Huff C, Hu H, Singleton M, Moore B, Xing J, et al. A probabilistic disease-gene finder for personal genomes. Genome Res. 2011;21(9):1529–42. 21700766 10.1101/gr.123158.111 PMC3166837

[223] Ye ZQ, Zhao SQ, Gao G, Liu XQ, Langlois RE, Lu H, et al. Finding new structural and sequence attributes to predict possible disease association of single amino acid polymorphism (SAP). Bioinformatics. 2007;23(12):1444–50. 17384424 10.1093/bioinformatics/btm119

[224] Yeo G, Burge CB. Maximum entropy modeling of short sequence motifs with applications to RNA splicing signals. J Comput Biol. 2004;11(2–3):377–94. 15285897 10.1089/1066527041410418

[225] Yin S, Ding F, Dokholyan NV. Modeling backbone flexibility improves protein stability estimation. Structure. 2007;15(12):1567–76. 18073107 10.1016/j.str.2007.09.024

[226] Yin S, Ding F, Dokholyan NV. Eris: an automated estimator of protein stability. Nat Methods. 2007;4(6):466–7. 17538626 10.1038/nmeth0607-466

[227] Yue P, Melamud E, Moult J. SNPs3D: candidate gene and SNP selection for association studies. BMC Bioinform. 2006;7:166. 10.1186/1471-2105-7-166 PMC1435944 16551372

[228] Yue P, Moult J. Identification and analysis of deleterious human SNPs. J Mol Biol. 2006;356(5):1263–74. 16412461 10.1016/j.jmb.2005.12.025

[229] Zambrano R, Jamroz M, Szczasiuk A, Pujols J, Kmiecik S, Ventura S. AGGRESCAN3D (A3D): server for prediction of aggregation properties of protein structures. Nucl Acids Res. 2015;43(W1):W306–13. 25883144 10.1093/nar/gkv359 PMC4489226

[230] Zeng S, Yang J, Chung BH, Lau YL, Yang W. EFIN: predicting the functional impact of nonsynonymous single nucleotide polymorphisms in human genome. BMC Genomics. 2014;15(1):455. 24916671 10.1186/1471-2164-15-455 PMC4061446

[231] Zhang C, Liu S, Zhu Q, Zhou Y. A knowledge-based energy function for protein–ligand, protein–protein, and protein–DNA complexes. J Med Chem. 2005;48(7):2325–35. 15801826 10.1021/jm049314d

[232] Zhang T, Wu Y, Lan Z, Shi Q, Yang Y, Guo J. Syntool: a novel region-based intolerance score to single nucleotide substitution for synonymous mutations predictions based on 123,136 individuals. Biomed Res Int. 2017;2017:5096208. 28812016 10.1155/2017/5096208 PMC5546077

[233] Zhao H, Yang Y, Lin H, Zhang X, Mort M, Cooper DN, et al. DDIG-in: discriminating between disease-associated and neutral non-frameshifting micro-indels. Genome Biol. 2013;14(3):R23. 23497682 10.1186/gb-2013-14-3-r23 PMC4053752

[234] Zhou H, Zhou Y. Distance-scaled, finite ideal-gas reference state improves structure-derived potentials of mean force for structure selection and stability prediction. Protein Sci. 2002;11(11):2714–26. 12381853 10.1110/ps.0217002 PMC2373736

[235] Zhou J, Theesfeld CL, Yao K, Chen KM, Wong AK, Troyanskaya OG. Deep learning sequence-based ab initio prediction of variant effects on expression and disease risk. Nat Genet. 2018;50(8):1171–9. 30013180 10.1038/s41588-018-0160-6 PMC6094955

[236] Zhou J, Troyanskaya OG. Predicting effects of noncoding variants with deep learning-based sequence model. Nat Methods. 2015;12(10):931–4. 26301843 10.1038/nmeth.3547 PMC4768299

[237] Addepalli A, Kalyani S, Singh M, Bandyopadhyay D, Mohan KN. CalPen (Calculator of Penetrance), a web-based tool to estimate penetrance in complex genetic disorders. PLoS ONE. 2020;15(1): e0228156. 31995602 10.1371/journal.pone.0228156 PMC6988981

[238] Alexander J, Mantzaris D, Georgitsi M, Drineas P, Paschou P. Variant Ranker: a web-tool to rank genomic data according to functional significance. BMC Bioinform. 2017;18(1):341. 10.1186/s12859-017-1752-3 PMC5514526 28716001

[239] Allot A, Peng Y, Wei CH, Lee K, Phan L, Lu Z. LitVar: a semantic search engine for linking genomic variant data in PubMed and PMC. Nucl Acids Res. 2018;46(W1):W530–6. 29762787 10.1093/nar/gky355 PMC6030971

[240] Ancien F, Pucci F, Godfroid M, Rooman M. Prediction and interpretation of deleterious coding variants in terms of protein structural stability. Sci Rep. 2018;8(1):4480. 29540703 10.1038/s41598-018-22531-2 PMC5852127

[241] Arani AA, Sehhati M, Tabatabaiefar MA. Genetic variant effect prediction by supervised nonnegative matrix tri-factorization. Mol Omics. 2021;17(5):740–51. 34164638 10.1039/D1MO00038A

[242] Avsec Z, Agarwal V, Visentin D, Ledsam JR, Grabska-Barwinska A, Taylor KR, et al. Effective gene expression prediction from sequence by integrating long-range interactions. Nat Methods. 2021;18(10):1196–203. 34608324 10.1038/s41592-021-01252-x PMC8490152

[243] Bailey M, Miller N. DMD Open-access Variant Explorer (DOVE): a scalable, open-access, web-based tool to aid in clinical interpretation of genetic variants in the DMD gene. Mol Genet Genomic Med. 2019;7(1): e00510. 30450799 10.1002/mgg3.510 PMC6382494

[244] Barbon L, Offord V, Radford EJ, Butler AP, Gerety SS, Adams DJ, et al. Variant Library Annotation Tool (VaLiAnT): an oligonucleotide library design and annotation tool for saturation genome editing and other deep mutational scanning experiments. Bioinformatics. 2022;38(4):892–9. 34791067 10.1093/bioinformatics/btab776 PMC8796380

[245] Basile AO, Byrska-Bishop M, Wallace J, Frase AT, Ritchie MD. Novel features and enhancements in BioBin, a tool for the biologically inspired binning and association analysis of rare variants. Bioinformatics. 2018;34(3):527–9. 28968757 10.1093/bioinformatics/btx559 PMC5860358

[246] Benegas G, Batra SS, Song YS. DNA language models are powerful predictors of genome-wide variant effects. Proc Natl Acad Sci USA. 2023;120(44): e2311219120. 37883436 10.1073/pnas.2311219120 PMC10622914

[247] Benton MC, Smith RA, Haupt LM, Sutherland HG, Dunn PJ, Albury CL, et al. Variant call format-diagnostic annotation and reporting tool: a customizable analysis pipeline for identification of clinically relevant genetic variants in next-generation sequencing data. J Mol Diagn. 2019;21(6):951–60. 31442673 10.1016/j.jmoldx.2019.07.001

[248] Bhattacharya S, Barseghyan H, Delot EC, Vilain E. nanotatoR: a tool for enhanced annotation of genomic structural variants. BMC Genomics. 2021;22(1):10. 33407088 10.1186/s12864-020-07182-w PMC7789800

[249] Binatti A, Bresolin S, Bortoluzzi S, Coppe A. iWhale: a computational pipeline based on Docker and SCons for detection and annotation of somatic variants in cancer WES data. Brief Bioinform. 2021;22(3):bbaa065. 32436933 10.1093/bib/bbaa065 PMC8557746

[250] Buniello A, MacArthur JAL, Cerezo M, Harris LW, Hayhurst J, Malangone C, et al. The NHGRI-EBI GWAS Catalog of published genome-wide association studies, targeted arrays and summary statistics 2019. Nucl Acids Res. 2019;47(D1):D1005–12. 30445434 10.1093/nar/gky1120 PMC6323933

[251] Calabrese R, Capriotti E, Fariselli P, Martelli PL, Casadio R. Functional annotations improve the predictive score of human disease-related mutations in proteins. Hum Mutat. 2009;30(8):1237–44. 19514061 10.1002/humu.21047

[252] Cao H, Wang J, He L, Qi Y, Zhang JZ. DeepDDG: predicting the stability change of protein point mutations using neural networks. J Chem Inf Model. 2019;59(4):1508–14. 30759982 10.1021/acs.jcim.8b00697

[253] Cao Y, Ha SY, So CC, Tong MT, Tang CS, Zhang H, et al. NGS4THAL, a one-stop molecular diagnosis and carrier screening tool for thalassemia and other hemoglobinopathies by next-generation sequencing. J Mol Diagn. 2022;24(10):1089–99.35868510 10.1016/j.jmoldx.2022.06.006

[254] Capriotti E, Fariselli P. PhD-SNPg: updating a webserver and lightweight tool for scoring nucleotide variants. Nucl Acids Res. 2023;51(W1):W451–8. 37246737 10.1093/nar/gkad455 PMC10320148

[255] Chakravarty D, Gao J, Phillips SM, Kundra R, Zhang H, Wang J, et al. OncoKB: a precision oncology knowledge base. JCO Precis Oncol. 2017. 10.1200/PO.17.0001. 28890946 10.1200/PO.17.0001 PMC5586540

[256] Chang MT, Bhattarai TS, Schram AM, Bielski CM, Donoghue MTA, Jonsson P, et al. Accelerating discovery of functional mutant alleles in cancer. Cancer Discov. 2018;8(2):174–83. 29247016 10.1158/2159-8290.CD-17-0321 PMC5809279

[257] Chen CW, Lin J, Chu YW. iStable: off-the-shelf predictor integration for predicting protein stability changes. BMC Bioinform. 2013;14 Suppl 2(Suppl 2):S5. 10.1186/1471-2105-14-S2-S5 PMC3549852 23369171

[258] Chen CW, Lin MH, Liao CC, Chang HP, Chu YW. iStable 2.0: predicting protein thermal stability changes by integrating various characteristic modules. Comput Struct Biotechnol J. 2020;18:622–30. 32226595 10.1016/j.csbj.2020.02.021 PMC7090336

[259] Chen Y, Lu H, Zhang N, Zhu Z, Wang S, Li M. PremPS: predicting the impact of missense mutations on protein stability. PLoS Comput Biol. 2020;16(12): e1008543. 33378330 10.1371/journal.pcbi.1008543 PMC7802934

[260] Cheng J, Novati G, Pan J, Bycroft C, Zemgulyte A, Applebaum T, et al. Accurate proteome-wide missense variant effect prediction with AlphaMissense. Science. 2023;381(6664):eadg7492. 37733863 10.1126/science.adg7492

[261] Chennen K, Weber T, Lornage X, Kress A, Bohm J, Thompson J, et al. MISTIC: A prediction tool to reveal disease-relevant deleterious missense variants. PLoS ONE. 2020;15(7): e0236962. 32735577 10.1371/journal.pone.0236962 PMC7394404

[262] Chun S, Fay JC. Identification of deleterious mutations within three human genomes. Genome Res. 2009;19(9):1553–61. 19602639 10.1101/gr.092619.109 PMC2752137

[263] Cipriani V, Pontikos N, Arno G, Sergouniotis PI, Lenassi E, Thawong P, et al. An improved phenotype-driven tool for rare mendelian variant prioritization: benchmarking exomiser on real patient whole-exome data. Genes. 2020;11(4):bbac176. 10.3390/genes11040460 PMC7230372 32340307

[264] Clausen R, Ma B, Nussinov R, Shehu A. Mapping the conformation space of wildtype and mutant H-ras with a memetic, cellular, and multiscale evolutionary algorithm. PLoS Comput Biol. 2015;11(9): e1004470. 26325505 10.1371/journal.pcbi.1004470 PMC4556523

[265] Cooper DN, Ball EV, Krawczak M. The human gene mutation database. Nucl Acids Res. 1998;26(1):285–7. 9399854 10.1093/nar/26.1.285 PMC147254

[266] Cooper DN, Stenson PD, Chuzhanova NA. The Human Gene Mutation Database (HGMD) and its exploitation in the study of mutational mechanisms. Curr Protoc Bioinformatics. 2006;Chapter 1:Unit 1 13. 10.1002/0471250953.bi0113s12 18428754

[267] Costanzo MC, Roselli C, Brandes M, Duby M, Hoang Q, Jang D, et al. Cardiovascular disease knowledge portal: a community resource for cardiovascular disease research. Circ Genom Precis Med. 2023;16(6): e004181. 37814896 10.1161/CIRCGEN.123.004181 PMC10843166

[268] Danis D, Jacobsen JOB, Balachandran P, Zhu Q, Yilmaz F, Reese J, et al. SvAnna: efficient and accurate pathogenicity prediction of coding and regulatory structural variants in long-read genome sequencing. Genome Med. 2022;14(1):44. 35484572 10.1186/s13073-022-01046-6 PMC9047340

[269] Danis D, Jacobsen JOB, Carmody LC, Gargano MA, McMurry JA, Hegde A, et al. Interpretable prioritization of splice variants in diagnostic next-generation sequencing. Am J Hum Genet. 2021;108(9):1564–77. 34289339 10.1016/j.ajhg.2021.06.014 PMC8456162

[270] Danzi MC, Dohrn MF, Fazal S, Beijer D, Rebelo AP, Cintra V, et al. Deep structured learning for variant prioritization in Mendelian diseases. Nat Commun. 2023;14(1):4167. 37443090 10.1038/s41467-023-39306-7 PMC10345112

[271] Derbel H, Zhao Z, Liu Q. Accurate prediction of functional effect of single amino acid variants with deep learning. Comput Struct Biotechnol J. 2023;21:5776–84. 38074467 10.1016/j.csbj.2023.11.017 PMC10709104

[272] Di Sera T, Velinder M, Ward A, Qiao Y, Georges S, Miller C, et al. Gene.iobio: an interactive web tool for versatile, clinically-driven variant interrogation and prioritization. Sci Rep. 2021;11(1):20307. 34645894 10.1038/s41598-021-99752-5 PMC8514592

[273] Dunham AS, Beltrao P, AlQuraishi M. High-throughput deep learning variant effect prediction with Sequence UNET. Genome Biol. 2023;24(1):110. 37161576 10.1186/s13059-023-02948-3 PMC10169183

[274] Ekawade A, Velinder M, Ward A, DiSera T, Miller C, Qiao Y, et al. Genepanel.iobio—an easy to use web tool for generating disease- and phenotype-associated gene lists. BMC Med Genomics. 2019;12(1):190. 31829207 10.1186/s12920-019-0641-1 PMC6907284

[275] Esposito D, Weile J, Shendure J, Starita LM, Papenfuss AT, Roth FP, et al. MaveDB: an open-source platform to distribute and interpret data from multiplexed assays of variant effect. Genome Biol. 2019;20(1):223. 31679514 10.1186/s13059-019-1845-6 PMC6827219

[276] Fang M, Su Z, Abolhassani H, Itan Y, Jin X, Hammarstrom L. VIPPID: a gene-specific single nucleotide variant pathogenicity prediction tool for primary immunodeficiency diseases. Brief Bioinform. 2022;23(5):bbac176. 35598327 10.1093/bib/bbac176 PMC9487673

[277] Feng BJ. PERCH: a unified framework for disease gene prioritization. Hum Mutat. 2017;38(3):243–51. 27995669 10.1002/humu.23158 PMC5299048

[278] Frazer J, Notin P, Dias M, Gomez A, Min JK, Brock K, et al. Disease variant prediction with deep generative models of evolutionary data. Nature. 2021;599(7883):91–5. 34707284 10.1038/s41586-021-04043-8

[279] Fredrich B, Schmohl M, Junge O, Gundlach S, Ellinghaus D, Pfeufer A, et al. VarWatch-A stand-alone software tool for variant matching. PLoS ONE. 2019;14(4): e0215618. 31022234 10.1371/journal.pone.0215618 PMC6483337

[280] Fu Y, Liu Z, Lou S, Bedford J, Mu XJ, Yip KY, et al. FunSeq2: a framework for prioritizing noncoding regulatory variants in cancer. Genome Biol. 2014;15(10):480. 25273974 10.1186/s13059-014-0480-5 PMC4203974

[281] Galano-Frutos JJ, Garcia-Cebollada H, Lopez A, Rosell M, de la Cruz X, Fernandez-Recio J, et al. PirePred: an accurate online consensus tool to interpret newborn screening-related genetic variants in structural context. J Mol Diagn. 2022;24(4):406–25. 35143952 10.1016/j.jmoldx.2022.01.005

[282] Ganel L, Abel HJ, FinMetSeq C, Hall IM. SVScore: an impact prediction tool for structural variation. Bioinformatics. 2017;33(7):1083–5. 28031184 10.1093/bioinformatics/btw789 PMC5408916

[283] Ganesan K, Kulandaisamy A, Binny Priya S, Gromiha MM. HuVarBase: a human variant database with comprehensive information at gene and protein levels. PLoS ONE. 2019;14(1): e0210475. 30703169 10.1371/journal.pone.0210475 PMC6354970

[284] Gao H, Hamp T, Ede J, Schraiber JG, McRae J, Singer-Berk M, et al. The landscape of tolerated genetic variation in humans and primates. Science. 2023;380(6648):eabn8153. 37262156 10.1126/science.abn8197 PMC10713091

[285] Gazzo AM, Daneels D, Cilia E, Bonduelle M, Abramowicz M, Van Dooren S, et al. DIDA: a curated and annotated digenic diseases database. Nucl Acids Res. 2016;44(D1):D900–7. 26481352 10.1093/nar/gkv1068 PMC4702791

[286] Geoffroy V, Pizot C, Redin C, Piton A, Vasli N, Stoetzel C, et al. VaRank: a simple and powerful tool for ranking genetic variants. PeerJ. 2015;3: e796. 25780760 10.7717/peerj.796 PMC4358652

[287] Glanzmann B, Herbst H, Kinnear CJ, Moller M, Gamieldien J, Bardien S. A new tool for prioritization of sequence variants from whole exome sequencing data. Source Code Biol Med. 2016;11:10. 27375772 10.1186/s13029-016-0056-8 PMC4929716

[288] Gong J, Wang J, Zong X, Ma Z, Xu D. Prediction of protein stability changes upon single-point variant using 3D structure profile. Comput Struct Biotechnol J. 2023;21:354–64. 36582438 10.1016/j.csbj.2022.12.008 PMC9791599

[289] Granata I, Sangiovanni M, Maiorano F, Miele M, Guarracino MR. Var2GO: a web-based tool for gene variants selection. BMC Bioinform. 2016;17(Suppl 12):376. 10.1186/s12859-016-1197-0 PMC5123234 28185576

[290] Griffith M, Spies NC, Krysiak K, McMichael JF, Coffman AC, Danos AM, et al. CIViC is a community knowledgebase for expert crowdsourcing the clinical interpretation of variants in cancer. Nat Genet. 2017;49(2):170–4. 28138153 10.1038/ng.3774 PMC5367263

[291] Guo Y, Tian K, Zeng H, Guo X, Gifford DK. A novel k-mer set memory (KSM) motif representation improves regulatory variant prediction. Genome Res. 2018;28(6):891–900. 29654070 10.1101/gr.226852.117 PMC5991515

[292] Gurbich TA, Ilinsky VV. ClassifyCNV: a tool for clinical annotation of copy-number variants. Sci Rep. 2020;10(1):20375. 33230148 10.1038/s41598-020-76425-3 PMC7683568

[293] Han Q, Yang Y, Wu S, Liao Y, Zhang S, Liang H, et al. Cruxome: a powerful tool for annotating, interpreting and reporting genetic variants. BMC Genomics. 2021;22(1):407. 34082700 10.1186/s12864-021-07728-6 PMC8173893

[294] Hart SN, Polley EC, Shimelis H, Yadav S, Couch FJ. Prediction of the functional impact of missense variants in BRCA1 and BRCA2 with BRCA-ML. NPJ Breast Cancer. 2020;6:13. 32377563 10.1038/s41523-020-0159-x PMC7190647

[295] He MM, Li Q, Yan M, Cao H, Hu Y, He KY, et al. Variant Interpretation for Cancer (VIC): a computational tool for assessing clinical impacts of somatic variants. Genome Med. 2019;11(1):53. 31443733 10.1186/s13073-019-0664-4 PMC6708137

[296] Howard M, Kane B, Lepry M, Stey P, Ragavendran A, Gamsiz Uzun ED. VarStack: a web tool for data retrieval to interpret somatic variants in cancer. Database. 2020;2020:baaa092. 33247936 10.1093/database/baaa092 PMC7698661

[297] Huang YF, Gulko B, Siepel A. Fast, scalable prediction of deleterious noncoding variants from functional and population genomic data. Nat Genet. 2017;49(4):618–24. 28288115 10.1038/ng.3810 PMC5395419

[298] Ip E, Chapman G, Winlaw D, Dunwoodie SL, Giannoulatou E. VPOT: a customizable variant prioritization ordering tool for annotated variants. Genomics Proteomics Bioinform. 2019;17(5):540–5. 10.1016/j.gpb.2019.11.001 PMC7056850 31765830

[299] Iqbal S, Hoksza D, Perez-Palma E, May P, Jespersen JB, Ahmed SS, et al. MISCAST: MIssense variant to protein StruCture Analysis web SuiTe. Nucl Acids Res. 2020;48(W1):W132–9. 32402084 10.1093/nar/gkaa361 PMC7319582

[300] Jagadeesh KA, Wenger AM, Berger MJ, Guturu H, Stenson PD, Cooper DN, et al. M-CAP eliminates a majority of variants of uncertain significance in clinical exomes at high sensitivity. Nat Genet. 2016;48(12):1581–6. 27776117 10.1038/ng.3703

[301] Jaganathan K, Kyriazopoulou Panagiotopoulou S, McRae JF, Darbandi SF, Knowles D, Li YI, et al. Predicting splicing from primary sequence with deep learning. Cell. 2019;176(3):535-48.e24. 30661751 10.1016/j.cell.2018.12.015

[302] Jagota M, Ye C, Albors C, Rastogi R, Koehl A, Ioannidis N, et al. Cross-protein transfer learning substantially improves disease variant prediction. Genome Biol. 2023;24(1):182. 37550700 10.1186/s13059-023-03024-6 PMC10408151

[303] Jiang S, Xie Y, He Z, Zhang Y, Zhao Y, Chen L, et al. m6ASNP: a tool for annotating genetic variants by m6A function. Gigascience. 2018;7(5):giy035. 29617790 10.1093/gigascience/giy035 PMC6007280

[304] Kaakinen M, Magi R, Fischer K, Heikkinen J, Jarvelin MR, Morris AP, et al. MARV: a tool for genome-wide multi-phenotype analysis of rare variants. BMC Bioinform. 2017;18(1):110. 10.1186/s12859-017-1530-2 PMC5311849 28209135

[305] Kalayci S, Selvan ME, Ramos I, Cotsapas C, Harris E, Kim EY, et al. ImmuneRegulation: a web-based tool for identifying human immune regulatory elements. Nucl Acids Res. 2019;47(W1):W142–50. 31114925 10.1093/nar/gkz450 PMC6602512

[306] Kamat MA, Blackshaw JA, Young R, Surendran P, Burgess S, Danesh J, et al. PhenoScanner V2: an expanded tool for searching human genotype-phenotype associations. Bioinformatics. 2019;35(22):4851–3. 31233103 10.1093/bioinformatics/btz469 PMC6853652

[307] Karmakar M, Cicaloni V, Rodrigues CHM, Spiga O, Santucci A, Ascher DB. HGDiscovery: an online tool providing functional and phenotypic information on novel variants of homogentisate 1,2-dioxigenase. Curr Res Struct Biol. 2022;4:271–7. 36118553 10.1016/j.crstbi.2022.08.001 PMC9471331

[308] Kasaragod S, Mohanty V, Tyagi A, Behera SK, Patil AH, Pinto SM, et al. CusVarDB: a tool for building customized sample-specific variant protein database from next-generation sequencing datasets. F1000Res. 2020;9:344. 33274046 10.12688/f1000research.23214.2 PMC7684676

[309] Katsonis P, Lichtarge O. A formal perturbation equation between genotype and phenotype determines the Evolutionary Action of protein-coding variations on fitness. Genome Res. 2014;24(12):2050–8. 25217195 10.1101/gr.176214.114 PMC4248321

[310] Krawczak M, Ball EV, Fenton I, Stenson PD, Abeysinghe S, Thomas N, et al. Human gene mutation database—a biomedical information and research resource. Hum Mutat. 2000;15(1):45–51. 10612821 10.1002/(SICI)1098-1004(200001)15:1<45::AID-HUMU10>3.0.CO;2-T

[311] Kulandaisamy A, Binny Priya S, Sakthivel R, Tarnovskaya S, Bizin I, Honigschmid P, et al. MutHTP: mutations in human transmembrane proteins. Bioinformatics. 2018;34(13):2325–6. 29401218 10.1093/bioinformatics/bty054

[312] Kulandaisamy A, Zaucha J, Frishman D, Gromiha MM. MPTherm-pred: analysis and prediction of thermal stability changes upon mutations in transmembrane proteins. J Mol Biol. 2021;433(11):166646. 32920050 10.1016/j.jmb.2020.09.005

[313] Laddach A, Gautel M, Fraternali F. TITINdb-a computational tool to assess titin’s role as a disease gene. Bioinformatics. 2017;33(21):3482–5. 29077808 10.1093/bioinformatics/btx424 PMC5860166

[314] Lai C, Zimmer AD, O’Connor R, Kim S, Chan R, van den Akker J, et al. LEAP: using machine learning to support variant classification in a clinical setting. Hum Mutat. 2020;41(6):1079–90. 32176384 10.1002/humu.24011 PMC7317941

[315] Lai J, Yang J, Gamsiz Uzun ED, Rubenstein BM, Sarkar IN. LYRUS: a machine learning model for predicting the pathogenicity of missense variants. Bioinform Adv. 2022;2(1):vbab045. 35036922 10.1093/bioadv/vbab045 PMC8754197

[316] Landrum MJ, Chitipiralla S, Brown GR, Chen C, Gu B, Hart J, et al. ClinVar: improvements to accessing data. Nucl Acids Res. 2020;48(D1):D835–44. 31777943 10.1093/nar/gkz972 PMC6943040

[317] Landrum MJ, Kattman BL. ClinVar at five years: delivering on the promise. Hum Mutat. 2018;39(11):1623–30. 30311387 10.1002/humu.23641 PMC11567647

[318] Landrum MJ, Lee JM, Benson M, Brown GR, Chao C, Chitipiralla S, et al. ClinVar: improving access to variant interpretations and supporting evidence. Nucl Acids Res. 2018;46(D1):D1062–7. 29165669 10.1093/nar/gkx1153 PMC5753237

[319] Lek M, Karczewski KJ, Minikel EV, Samocha KE, Banks E, Fennell T, et al. Analysis of protein-coding genetic variation in 60,706 humans. Nature. 2016;536(7616):285–91. 27535533 10.1038/nature19057 PMC5018207

[320] Leman R, Gaildrat P, Le Gac G, Ka C, Fichou Y, Audrezet MP, et al. Novel diagnostic tool for prediction of variant spliceogenicity derived from a set of 395 combined in silico/in vitro studies: an international collaborative effort. Nucl Acids Res. 2018;46(15):7913–23. 29750258 10.1093/nar/gky372 PMC6125621

[321] Leman R, Harter V, Atkinson A, Davy G, Rousselin A, Muller E, et al. SpliceLauncher: a tool for detection, annotation and relative quantification of alternative junctions from RNAseq data. Bioinformatics. 2020;36(5):1634–6. 31617569 10.1093/bioinformatics/btz784

[322] Leman R, Parfait B, Vidaud D, Girodon E, Pacot L, Le Gac G, et al. SPiP: Splicing Prediction Pipeline, a machine learning tool for massive detection of exonic and intronic variant effects on mRNA splicing. Hum Mutat. 2022;43(12):2308–23. 36273432 10.1002/humu.24491 PMC10946553

[323] Leslie R, O’Donnell CJ, Johnson AD. GRASP: analysis of genotype-phenotype results from 1390 genome-wide association studies and corresponding open access database. Bioinformatics. 2014;30(12):i185–94. 24931982 10.1093/bioinformatics/btu273 PMC4072913

[324] Li C, Zhi D, Wang K, Liu X. MetaRNN: differentiating rare pathogenic and rare benign missense SNVs and InDels using deep learning. Genome Med. 2022;14(1):115. 36209109 10.1186/s13073-022-01120-z PMC9548151

[325] Li G, Pahari S, Murthy AK, Liang S, Fragoza R, Yu H, et al. SAAMBE-SEQ: a sequence-based method for predicting mutation effect on protein-protein binding affinity. Bioinformatics. 2021;37(7):992–9. 32866236 10.1093/bioinformatics/btaa761 PMC8128451

[326] Li G, Panday SK, Alexov E. SAAFEC-SEQ: a sequence-based method for predicting the effect of single point mutations on protein thermodynamic stability. Int J Mol Sci. 2021;22(2):606. 33435356 10.3390/ijms22020606 PMC7827184

[327] Li G, Yao S, Fan L. ProSTAGE: predicting effects of mutations on protein stability by using protein embeddings and graph convolutional networks. J Chem Inf Model. 2024;64(2):340–7. 38166383 10.1021/acs.jcim.3c01697 PMC10806799

[328] Li H, Liu S, Wang S, Zeng Q, Chen Y, Fang T, et al. Cancer SIGVAR: a semiautomated interpretation tool for germline variants of hereditary cancer-related genes. Hum Mutat. 2021;42(4):359–72. 33565189 10.1002/humu.24177

[329] Li M, Simonetti FL, Goncearenco A, Panchenko AR. MutaBind estimates and interprets the effects of sequence variants on protein-protein interactions. Nucl Acids Res. 2016;44(W1):W494-501. 27150810 10.1093/nar/gkw374 PMC4987923

[330] Liu Y, Dougherty JD. utr.annotation: a tool for annotating genomic variants that could influence post-transcriptional regulation. Bioinformatics. 2021;37(21):3926–8. 34478494 10.1093/bioinformatics/btab635 PMC10186104

[331] Lott MT, Leipzig JN, Derbeneva O, Xie HM, Chalkia D, Sarmady M, et al. mtDNA variation and analysis using mitomap and mitomaster. Curr Protoc Bioinform. 2013;44(123):1–6. 10.1002/0471250953.bi0123s44 PMC4257604 25489354

[332] Lou S, Cotter KA, Li T, Liang J, Mohsen H, Liu J, et al. GRAM: A GeneRAlized Model to predict the molecular effect of a non-coding variant in a cell-type specific manner. PLoS Genet. 2019;15(8): e1007860. 31469829 10.1371/journal.pgen.1007860 PMC6742416

[333] Lu H, Ma L, Quan C, Li L, Lu Y, Zhou G, et al. RegVar: tissue-specific prioritization of non-coding regulatory variants. Genomics Proteomics Bioinform. 2023;21(2):385–95. 10.1016/j.gpb.2021.08.011 PMC10626172 34973416

[334] Malhis N, Jacobson M, Jones SJM, Gsponer J. LIST-S2: taxonomy based sorting of deleterious missense mutations across species. Nucl Acids Res. 2020;48(W1):W154–61. 32352516 10.1093/nar/gkaa288 PMC7319545

[335] Malhis N, Jones SJM, Gsponer J. Improved measures for evolutionary conservation that exploit taxonomy distances. Nat Commun. 2019;10(1):1556. 30952844 10.1038/s41467-019-09583-2 PMC6450959

[336] Markham JF, Yerneni S, Ryland GL, Leong HS, Fellowes A, Thompson ER, et al. CNspector: a web-based tool for visualisation and clinical diagnosis of copy number variation from next generation sequencing. Sci Rep. 2019;9(1):6426. 31015508 10.1038/s41598-019-42858-8 PMC6478945

[337] Marquet C, Heinzinger M, Olenyi T, Dallago C, Erckert K, Bernhofer M, et al. Embeddings from protein language models predict conservation and variant effects. Hum Genet. 2022;141(10):1629–47. 34967936 10.1007/s00439-021-02411-y PMC8716573

[338] Martin-Antoniano I, Alonso L, Madrid M, Lopez de Maturana E, Malats N. DoriTool: a bioinformatics integrative tool for post-association functional annotation. Public Health Genom. 2017;20(2):126–35. 10.1159/000477561 28700989

[339] McVicker G, Gordon D, Davis C, Green P. Widespread genomic signatures of natural selection in hominid evolution. PLoS Genet. 2009;5(5): e1000471. 19424416 10.1371/journal.pgen.1000471 PMC2669884

[340] Menon R, Patel NV, Mohapatra A, Joshi CG. VDAP-GUI: a user-friendly pipeline for variant discovery and annotation of raw next-generation sequencing data. 3 Biotech. 2016;6(1):68. 28330138 10.1007/s13205-016-0382-1 PMC4754298

[341] Montanucci L, Capriotti E, Frank Y, Ben-Tal N, Fariselli P. DDGun: an untrained method for the prediction of protein stability changes upon single and multiple point variations. BMC Bioinform. 2019;20(Suppl 14):335. 10.1186/s12859-019-2923-1 PMC6606456 31266447

[342] Munro D, Singh M. DeMaSk: a deep mutational scanning substitution matrix and its use for variant impact prediction. Bioinformatics. 2021;36(22–23):5322–9. 33325500 10.1093/bioinformatics/btaa1030 PMC8016454

[343] Nachtegael C, Gravel B, Dillen A, Smits G, Nowe A, Papadimitriou S, et al. Scaling up oligogenic diseases research with OLIDA: the Oligogenic Diseases Database. Database. 2022;2–22:baac023. 10.1093/database/baac023 PMC9216476 35411390

[344] Ng PC, Henikoff S. Predicting deleterious amino acid substitutions. Genome Res. 2001;11(5):863–74. 11337480 10.1101/gr.176601 PMC311071

[345] Nishio SY, Usami SI. The clinical next-generation sequencing database: a tool for the unified management of clinical information and genetic variants to accelerate variant pathogenicity classification. Hum Mutat. 2017;38(3):252–9. 28008688 10.1002/humu.23160 PMC5324660

[346] Pagel KA, Antaki D, Lian A, Mort M, Cooper DN, Sebat J, et al. Pathogenicity and functional impact of non-frameshifting insertion/deletion variation in the human genome. PLoS Comput Biol. 2019;15(6): e1007112. 31199787 10.1371/journal.pcbi.1007112 PMC6594643

[347] Pahari S, Li G, Murthy AK, Liang S, Fragoza R, Yu H, et al. SAAMBE-3D: predicting effect of mutations on protein-protein interactions. Int J Mol Sci. 2020;21(7):2563. 32272725 10.3390/ijms21072563 PMC7177817

[348] Pais LS, Snow H, Weisburd B, Zhang S, Baxter SM, DiTroia S, et al. seqr: a web-based analysis and collaboration tool for rare disease genomics. Hum Mutat. 2022;43(6):698–707. 35266241 10.1002/humu.24366 PMC9903206

[349] Palheta HGA, Goncalves WG, Brito LM, Dos Santos AR, Dos Reis Matsumoto M, Ribeiro-Dos-Santos A, et al. AmazonForest: in silico metaprediction of pathogenic variants. Biology. 2022;11(4):538. 35453737 10.3390/biology11040538 PMC9024711

[350] Pancotti C, Benevenuta S, Repetto V, Birolo G, Capriotti E, Sanavia T, et al. A deep-learning sequence-based method to predict protein stability changes upon genetic variations. Genes (Basel). 2021;12(6):911. 34204764 10.3390/genes12060911 PMC8231498

[351] Pei G, Hu R, Jia P, Zhao Z. DeepFun: a deep learning sequence-based model to decipher non-coding variant effect in a tissue- and cell type-specific manner. Nucleic Acids Res. 2021;49(W1):W131–9. 34048560 10.1093/nar/gkab429 PMC8262726

[352] Petukh M, Dai L, Alexov E. SAAMBE: webserver to predict the charge of binding free energy caused by amino acids mutations. Int J Mol Sci. 2016;17(4):547. 27077847 10.3390/ijms17040547 PMC4849003

[353] Pires DE, Ascher DB, Blundell TL. mCSM: predicting the effects of mutations in proteins using graph-based signatures. Bioinformatics. 2014;30(3):335–42. 24281696 10.1093/bioinformatics/btt691 PMC3904523

[354] Piriyapongsa J, Sukritha C, Kaewprommal P, Intarat C, Triparn K, Phornsiricharoenphant K, et al. PharmVIP: a web-based tool for pharmacogenomic variant analysis and interpretation. J Pers Med. 2021;11(11):1230. 34834582 10.3390/jpm11111230 PMC8618518

[355] Ponzoni L, Penaherrera DA, Oltvai ZN, Bahar I. Rhapsody: predicting the pathogenicity of human missense variants. Bioinformatics. 2020;36(10):3084–92. 32101277 10.1093/bioinformatics/btaa127 PMC7214033

[356] Popov P, et al. Prediction of disease-associated mutations in the transmembrane regions of proteins with known 3D structure. PLoS ONE. 2019;14(7):e0219452. 31291347 10.1371/journal.pone.0219452 PMC6620012

[357] Prive F, Albinana C, Arbel J, Pasaniuc B, Vilhjalmsson BJ. Inferring disease architecture and predictive ability with LDpred2-auto. Am J Hum Genet. 2023;110(12):2042–55. 37944514 10.1016/j.ajhg.2023.10.010 PMC10716363

[358] Prunier J, Lemacon A, Bastien A, Jafarikia M, Porth I, Robert C, et al. LD-annot: a bioinformatics tool to automatically provide candidate SNPs with annotations for genetically linked genes. Front Genet. 2019;10:1192. 31850063 10.3389/fgene.2019.01192 PMC6889475

[359] Qi H, Zhang H, Zhao Y, Chen C, Long JJ, Chung WK, et al. MVP predicts the pathogenicity of missense variants by deep learning. Nat Commun. 2021;12(1):510. 33479230 10.1038/s41467-020-20847-0 PMC7820281

[360] Quan L, Lv Q, Zhang Y. STRUM: structure-based prediction of protein stability changes upon single-point mutation. Bioinformatics. 2016;32(19):2936–46. 27318206 10.1093/bioinformatics/btw361 PMC5039926

[361] Quinodoz M, Peter VG, Bedoni N, Royer Bertrand B, Cisarova K, Salmaninejad A, et al. AutoMap is a high performance homozygosity mapping tool using next-generation sequencing data. Nat Commun. 2021;12(1):518. 33483490 10.1038/s41467-020-20584-4 PMC7822856

[362] Quinones-Valdez G, Fu T, Chan TW, Xiao X. scAllele: a versatile tool for the detection and analysis of variants in scRNA-seq. Sci Adv. 2022;8(35):eabn6398. 36054357 10.1126/sciadv.abn6398 PMC11636672

[363] Radusky L, Modenutti C, Delgado J, Bustamante JP, Vishnopolska S, Kiel C, et al. VarQ: a tool for the structural and functional analysis of human protein variants. Front Genet. 2018;9:620. 30574164 10.3389/fgene.2018.00620 PMC6291447

[364] Raimondi D, Gazzo AM, Rooman M, Lenaerts T, Vranken WF. Multilevel biological characterization of exomic variants at the protein level significantly improves the identification of their deleterious effects. Bioinformatics. 2016;32(12):1797–804. 27153718 10.1093/bioinformatics/btw094

[365] Raimondi D, Tanyalcin I, Ferte J, Gazzo A, Orlando G, Lenaerts T, et al. DEOGEN2: prediction and interactive visualization of single amino acid variant deleteriousness in human proteins. Nucl Acids Res. 2017;45(W1):W201–6. 28498993 10.1093/nar/gkx390 PMC5570203

[366] Rastogi R, Stenson PD, Cooper DN, Bejerano G. X-CAP improves pathogenicity prediction of stopgain variants. Genome Med. 2022;14(1):81. 35906703 10.1186/s13073-022-01078-y PMC9338606

[367] Rathinakannan VS, Schukov HP, Heron S, Schleutker J, Sipeky C. ShAn: an easy-to-use tool for interactive and integrated variant annotation. PLoS ONE. 2020;15(7): e0235669. 32634151 10.1371/journal.pone.0235669 PMC7340278

[368] Ravichandran V, Shameer Z, Kemel Y, Walsh M, Cadoo K, Lipkin S, et al. Toward automation of germline variant curation in clinical cancer genetics. Genet Med. 2019;21(9):2116–25. 30787465 10.1038/s41436-019-0463-8 PMC6703969

[369] Rehm HL, Berg JS, Brooks LD, Bustamante CD, Evans JP, Landrum MJ, et al. ClinGen–the clinical genome resource. N Engl J Med. 2015;372(23):2235–42. 26014595 10.1056/NEJMsr1406261 PMC4474187

[370] Rentzsch P, Witten D, Cooper GM, Shendure J, Kircher M. CADD: predicting the deleteriousness of variants throughout the human genome. Nucl Acids Res. 2019;47(D1):D886–94. 30371827 10.1093/nar/gky1016 PMC6323892

[371] Rives A, Meier J, Sercu T, Goyal S, Lin Z, Liu J, et al. Biological structure and function emerge from scaling unsupervised learning to 250 million protein sequences. Proc Natl Acad Sci USA. 2021;118(15):e2016239118. 33876751 10.1073/pnas.2016239118 PMC8053943

[372] Rodrigues CH, Pires DE, Ascher DB. DynaMut: predicting the impact of mutations on protein conformation, flexibility and stability. Nucl Acids Res. 2018;46(W1):W350–5. 29718330 10.1093/nar/gky300 PMC6031064

[373] Rodrigues CHM, Pires DEV, Ascher DB. DynaMut2: assessing changes in stability and flexibility upon single and multiple point missense mutations. Protein Sci. 2021;30(1):60–9. 32881105 10.1002/pro.3942 PMC7737773

[374] Rogers MF, Shihab HA, Gaunt TR, Campbell C. CScape: a tool for predicting oncogenic single-point mutations in the cancer genome. Sci Rep. 2017;7(1):11597. 28912487 10.1038/s41598-017-11746-4 PMC5599557

[375] Rogers MF, Shihab HA, Mort M, Cooper DN, Gaunt TR, Campbell C. FATHMM-XF: accurate prediction of pathogenic point mutations via extended features. Bioinformatics. 2018;34(3):511–3. 28968714 10.1093/bioinformatics/btx536 PMC5860356

[376] Sasorith S, Baux D, Bergougnoux A, Paulet D, Lahure A, Bareil C, et al. The CYSMA web server: an example of integrative tool for in silico analysis of missense variants identified in Mendelian disorders. Hum Mutat. 2020;41(2):375–86. 31674704 10.1002/humu.23941

[377] Seva J, Wiegandt DL, Gotze J, Lamping M, Rieke D, Schafer R, et al. VIST—a variant-information search tool for precision oncology. BMC Bioinform. 2019;20(1):429. 10.1186/s12859-019-2958-3 PMC6697931 31419935

[378] Shamsi Z, Chan M, Shukla D. TLmutation: predicting the effects of mutations using transfer learning. J Phys Chem B. 2020;124(19):3845–54. 32308006 10.1021/acs.jpcb.0c00197

[379] Sharo AG, Hu Z, Sunyaev SR, Brenner SE. StrVCTVRE: a supervised learning method to predict the pathogenicity of human genome structural variants. Am J Hum Genet. 2022;109(2):195–209. 35032432 10.1016/j.ajhg.2021.12.007 PMC8874149

[380] Shibata A, Okuno T, Rahman MA, Azuma Y, Takeda J, Masuda A, et al. IntSplice: prediction of the splicing consequences of intronic single-nucleotide variations in the human genome. J Hum Genet. 2016;61(7):633–40. 27009626 10.1038/jhg.2016.23

[381] Shin J, Jeon J, Jung D, Kim K, Kim YJ, Jeong DH, et al. PhenGenVar: a user-friendly genetic variant detection and visualization tool for precision medicine. J Pers Med. 2022;12(6):959. 35743744 10.3390/jpm12060959 PMC9224645

[382] Sokolova K, Theesfeld CL, Wong AK, Zhang Z, Dolinski K, Troyanskaya OG. Atlas of primary cell-type-specific sequence models of gene expression and variant effects. Cell Rep Methods. 2023;3(9): 100580. 37703883 10.1016/j.crmeth.2023.100580 PMC10545936

[383] Spector JD, Wiita AP. ClinTAD: a tool for copy number variant interpretation in the context of topologically associated domains. J Hum Genet. 2019;64(5):437–43. 30765865 10.1038/s10038-019-0573-9

[384] Staley JR, Blackshaw J, Kamat MA, Ellis S, Surendran P, Sun BB, et al. PhenoScanner: a database of human genotype-phenotype associations. Bioinformatics. 2016;32(20):3207–9. 27318201 10.1093/bioinformatics/btw373 PMC5048068

[385] Steinhaus R, Proft S, Schuelke M, Cooper DN, Schwarz JM, Seelow D. MutationTaster2021. Nucl Acids Res. 2021;49(W1):W446–51. 33893808 10.1093/nar/gkab266 PMC8262698

[386] Stenson PD, Ball EV, Mort M, Phillips AD, Shaw K, Cooper DN. The Human Gene Mutation Database (HGMD) and its exploitation in the fields of personalized genomics and molecular evolution. Curr Protoc Bioinform. 2012;Chapter 1:1 13 1–1 20 10.1002/0471250953.bi0113s39 22948725

[387] Stenson PD, Ball EV, Mort M, Phillips AD, Shiel JA, Thomas NS, et al. Human Gene Mutation Database (HGMD): 2003 update. Hum Mutat. 2003;21(6):577–81. 12754702 10.1002/humu.10212

[388] Stenson PD, Mort M, Ball EV, Chapman M, Evans K, Azevedo L, et al. The Human Gene Mutation Database (HGMD((R))): optimizing its use in a clinical diagnostic or research setting. Hum Genet. 2020;139(10):1197–207. 32596782 10.1007/s00439-020-02199-3 PMC7497289

[389] Stenson PD, Mort M, Ball EV, Howells K, Phillips AD, Thomas NS, et al. The human gene mutation database: 2008 update. Genome Med. 2009;1(1):13. 19348700 10.1186/gm13 PMC2651586

[390] Stenson PD, Mort M, Ball EV, Shaw K, Phillips A, Cooper DN. The Human Gene Mutation Database: building a comprehensive mutation repository for clinical and molecular genetics, diagnostic testing and personalized genomic medicine. Hum Genet. 2014;133(1):1–9. 24077912 10.1007/s00439-013-1358-4 PMC3898141

[391] Sun W, Duan T, Ye P, Chen K, Zhang G, Lai M, et al. TSVdb: a web-tool for TCGA splicing variants analysis. BMC Genomics. 2018;19(1):405. 29843604 10.1186/s12864-018-4775-x PMC5975414

[392] Sundaram L, Gao H, Padigepati SR, McRae JF, Li Y, Kosmicki JA, et al. Predicting the clinical impact of human mutation with deep neural networks. Nat Genet. 2018;50(8):1161–70. 30038395 10.1038/s41588-018-0167-z PMC6237276

[393] Takata A, Hamanaka K, Matsumoto N. Refinement of the clinical variant interpretation framework by statistical evidence and machine learning. Med. 2021;2(5):611-32.e9. 35590234 10.1016/j.medj.2021.02.003

[394] Takeda JI, Fukami S, Tamura A, Shibata A, Ohno K. IntSplice2: prediction of the splicing effects of intronic single-nucleotide variants using LightGBM modeling. Front Genet. 2021;12: 701076. 34349788 10.3389/fgene.2021.701076 PMC8326971

[395] Takeda JI, Nanatsue K, Yamagishi R, Ito M, Haga N, Hirata H, et al. InMeRF: prediction of pathogenicity of missense variants by individual modeling for each amino acid substitution. NAR Genom Bioinform. 2020;2(2):lqaa038. 33543123 10.1093/nargab/lqaa038 PMC7671370

[396] Tamborero D, Rubio-Perez C, Deu-Pons J, Schroeder MP, Vivancos A, Rovira A, et al. Cancer genome interpreter annotates the biological and clinical relevance of tumor alterations. Genome Med. 2018;10(1):25. 29592813 10.1186/s13073-018-0531-8 PMC5875005

[397] Thanapattheerakul T, Engchuan W, Chan JH. Predicting the effect of variants on splicing using convolutional neural networks. PeerJ. 2020;8: e9470. 32704450 10.7717/peerj.9470 PMC7346860

[398] Thornton AM, Fang L, Lo A, McSharry M, Haan D, O’Brien C, et al. eVIP2: expression-based variant impact phenotyping to predict the function of gene variants. PLoS Comput Biol. 2021;17(7): e1009132. 34214079 10.1371/journal.pcbi.1009132 PMC8281988

[399] Tokheim C, Karchin R. CHASMplus reveals the scope of somatic missense mutations driving human cancers. Cell Syst. 2019;9(1):9-23.e8. 31202631 10.1016/j.cels.2019.05.005 PMC6857794

[400] Tong SY, Fan K, Zhou ZW, Liu LY, Zhang SQ, Fu Y, et al. mvPPT: a highly efficient and sensitive pathogenicity prediction tool for missense variants. Genomics Proteomics Bioinform. 2023;21(2):414–26. 10.1016/j.gpb.2022.07.005 PMC10626173 35940520

[401] Trovato A, Seno F, Tosatto SC. The PASTA server for protein aggregation prediction. Protein Eng Des Sel. 2007;20(10):521–3. 17720750 10.1093/protein/gzm042

[402] Turner TN, et al. denovo-db: a compendium of human de novo variants. Nucl Acids Res. 2017;45(D1):D804–11. 27907889 10.1093/nar/gkw865 PMC5210614

[403] Wang J, Liu Z, Bellen HJ, Yamamoto S. Navigating MARRVEL, a web-based tool that integrates human genomics and model organism genetics information. J Vis Exp. 2019;150:e59542. 10.3791/59542 PMC7401700 31475990

[404] Wang M, Deng W, Samuels DC, Zhao Z, Simon LM. MitoTrace: a computational framework for analyzing mitochondrial variation in single-cell RNA sequencing data. Genes (Basel). 2023;14(6):1222. 37372402 10.3390/genes14061222 PMC10298143

[405] Ward LD, Kellis M. HaploReg: a resource for exploring chromatin states, conservation, and regulatory motif alterations within sets of genetically linked variants. Nucl Acids Res. 2012;40(Database issue):D930–4. 22064851 10.1093/nar/gkr917 PMC3245002

[406] Wells A, Heckerman D, Torkamani A, Yin L, Sebat J, Ren B, et al. Ranking of non-coding pathogenic variants and putative essential regions of the human genome. Nat Commun. 2019;10(1):5241. 31748530 10.1038/s41467-019-13212-3 PMC6868241

[407] Won DG, Kim DW, Woo J, Lee K. 3Cnet: pathogenicity prediction of human variants using multitask learning with evolutionary constraints. Bioinformatics. 2021;37(24):4626–34. 34270679 10.1093/bioinformatics/btab529 PMC8665754

[408] Woodard J, Zhang C, Zhang Y. ADDRESS: a database of disease-associated human variants incorporating protein structure and folding stabilities. J Mol Biol. 2021;433(11): 166840. 33539887 10.1016/j.jmb.2021.166840 PMC8119349

[409] Wu Y, Li R, Sun S, Weile J, Roth FP. Improved pathogenicity prediction for rare human missense variants. Am J Hum Genet. 2021;108(10):1891–906. 34551312 10.1016/j.ajhg.2021.08.012 PMC8546039

[410] Xavier A, Scott RJ, Talseth-Palmer BA. TAPES: a tool for assessment and prioritisation in exome studies. PLoS Comput Biol. 2019;15(10): e1007453. 31613886 10.1371/journal.pcbi.1007453 PMC6814239

[411] Xiang J, Peng J, Baxter S, Peng Z. AutoPVS1: an automatic classification tool for PVS1 interpretation of null variants. Hum Mutat. 2020;41(9):1488–98. 32442321 10.1002/humu.24051

[412] Xiao Y, Wang J, Li J, Zhang P, Li J, Zhou Y, et al. An analytical framework for decoding cell type-specific genetic variation of gene regulation. Nat Commun. 2023;14(1):3884. 37391400 10.1038/s41467-023-39538-7 PMC10313894

[413] Yue Z, Zhao L, Cheng N, Yan H, Xia J. dbCID: a manually curated resource for exploring the driver indels in human cancer. Brief Bioinform. 2019;20(5):1925–33. 30016397 10.1093/bib/bby059

[414] Zhang H, Xu MS, Fan X, Chung WK, Shen Y. Predicting functional effect of missense variants using graph attention neural networks. Nat Mach Intell. 2022;4(11):1017–28. 37484202 10.1038/s42256-022-00561-w PMC10361701

[415] Zhang N, Chen Y, Lu H, Zhao F, Alvarez RV, Goncearenco A, et al. MutaBind2: predicting the impacts of single and multiple mutations on protein-protein interactions. iScience. 2020;23(3):100939. 32169820 10.1016/j.isci.2020.100939 PMC7068639

[416] Zhang X, Walsh R, Whiffin N, Buchan R, Midwinter W, Wilk A, et al. Disease-specific variant pathogenicity prediction significantly improves variant interpretation in inherited cardiac conditions. Genet Med. 2021;23(1):69–79. 33046849 10.1038/s41436-020-00972-3 PMC7790749

[417] Zhou J, Gao J, Zhang H, Zhao D, Li A, Iqbal F, et al. PedMiner: a tool for linkage analysis-based identification of disease-associated variants using family based whole-exome sequencing data. Brief Bioinform. 2021;22(3):bbaa077. 32393981 10.1093/bib/bbaa077 PMC8138824

[418] Zia M, Spurgeon P, Levesque A, Furlani T, Wang J. GenESysV: a fast, intuitive and scalable genome exploration open source tool for variants generated from high-throughput sequencing projects. BMC Bioinform. 2019;20(1):61. 10.1186/s12859-019-2636-5 PMC6357466 30704396

[419] Aoki E, Manabe N, Ohno S, Aoki T, Furukawa JI, Togayachi A, et al. Predicting the pathogenicity of missense variants based on protein instability to support diagnosis of patients with novel variants of ARSL. Mol Genet Metab Rep. 2023;37: 101016. 38053926 10.1016/j.ymgmr.2023.101016 PMC10694752

[420] Dereli O, Kuru N, Akkoyun E, Bircan A, Tastan O, Adebali O. PHACTboost: a phylogeny-aware pathogenicity predictor for missense mutations via boosting. Mol Biol Evol. 2024;41(7):msae136. 38934805 10.1093/molbev/msae136 PMC11251492

[421] Kuru N, Dereli O, Akkoyun E, Bircan A, Tastan O, Adebali O. PHACT: phylogeny-aware computing of tolerance for missense mutations. Mol Biol Evol. 2022;39(6):msac114. 35639618 10.1093/molbev/msac114 PMC9178230

[422] Rastogi R, Chung R, Li S, Li C, Lee K, Woo J, et al. Critical assessment of missense variant effect predictors on disease-relevant variant data. bioRxiv. 2024. 10.1101/2024.06.06.597828. 39091757 10.1101/2024.06.06.597828 PMC11291157

