# Supplementary material for: Variant Impact Predictor database (VIPdb), version 2: trends from three decades of genetic variant impact predictors
Source: Hum Genomics. 2024 Aug 28;18:90. doi: 10.1186/s40246-024-00663-z (PMC11360829; doi:10.1186/s40246-024-00663-z)
Supplement: Supplementary file 2 — Supplementary Material 2. [file 40246_2024_663_MOESM2_ESM.pdf]

VIPdb version 2.01 field description for VIPdb_v2.01.csv

VIPdb metadata

Ref_ID	VIPdb reference ID. Each unique PubMed ID has a unique reference ID.
VIP_ID	VIPdb VIP ID. Each unique VIP name has a unique VIP ID.
VIPdb_version	Number indicating if the VIP is curated in VIPdb version 1 or version 2
VIP_name	The name of the VIP
VIP_family_name	VIP family name used to group VIPs for citation analyses
Primarily_for_VIP	Binary value indicating if the VIP is primarily designed for predicting variant impact. VIPs not originally designed for variant impact prediction but nonetheless used for this purpose, such as those estimating conservation scores and population allele frequencies, were categorized as non-primary.
Database	Binary value indicating if the VIP is a database

Variant type

SNVs	Binary value indicating if the VIP addresses single nucleotide variants
Indel	Binary value indicating if the VIP addresses small insertions and deletions
SVs	Binary value indicating if a VIP addresses structural variants
Nonsynonymous_ nonsense	Binary value indicating if the VIP addresses nonsynonymous and/or nonsense variants
Synonymous	Binary value indicating if the VIP addresses synonymous variants
Splicing	Binary value indicating if the VIP addresses splicing variants
Regulatory_regions	Binary value indicating if the VIP addresses other regulatory variants, such as variants in enhancer regions
Gene-specific	List of genes if the VIP is designed for specific genes
Somatic	Binary value indicating if the VIP addresses somatic variants

ClinGen SVI calibration

ClinGen_SVI_ calibration	Binary value indicating if the VIP was calibrated with ClinGen SVI ACMG/AMP guidelines in Pejaver et al., 2022
Higher_score_ greater_pathogenicity	Binary value indicating if higher scores from the VIP indicate greater pathogenicity of the variant. If yes (1), scores <= for BP4 thresholds and >= for PB3 thresholds are used. If no (0), scores >= for BP4 thresholds and <= for PB3 thresholds are used. For example, with REVEL, where this is true (1), variants with scores <= 0.003 are classified as BP4 with -8 points, and scores between (0.03, 0.016] are clssified as BP4 with -4 points. Conversely, variants with scores >=-0.932 are classified as PP3 with +4 poins, and scores in the range [0.773, 0.932) are classified as PP3 with +2 points.
BP4_-8pts	Threshold for BP4 negative eight points
BP4_-4pts	Threshold for BP4 negative four points
BP4_-2pts	Threshold for BP4 negative two points
BP4_-1pts	Threshold for BP4 negative one point
PP3_+1pt	Threshold for PP3 positive one point

PP3_+2pt	Threshold for PP3 positive two points
PP3_+4pt	Threshold for PP3 positive four points
PP3_+8pt	Threshold for PP3 positive eight points

CAGI Annotate All Missense / Missense Marathon (AAMMM)

CAGI_AAMMM	Binary value indicating if the VIP was assessed in CAGI Annotate-All-Missense / Missense Marathon (AAMMM) challenge in Rastogi et al., 2024
CAGI_AAMMM_ predictor	The annotation used in CAGI_AAMMM paper (Rastogi et al., 2024)
AAMMM_AUROC_ full-dataset	Area under the receiver operating characteristic curve for the full dataset
AAMMM_AUROC_ high-specificity	AUROC for the high-specificity area (FPR <= 5%)
AAMMM_AUROC_ high-sensitivity	AUROC for the high-sensitivity area (TPR >= 95%)

dbNSFP

dbNSFP_Academic	Binary value indicating if the VIP is within dbNSFP4.7a academic database
dbNSFP_Commercial	Binary value indicating if the VIP is within dbNSFP4.7c commercial database

License

License_academic	Binary value indicating if the VIP is free for academic use
License_commercial	Binary value indicating if the VIP is free for commercial use
License_description	Description of the VIP license

Accessibility

Downloadable_pre-calculated_annotation	Binary value indicating if pre-calculated annotations or scores are available
Standalone	Binary value indicating if a stand-alone version of the VIP is available
Web_server	Binary value indicating if a web server for the VIP is available
Website_accessible	Binary value indicating the accessibility of the VIP's homepage
Homepage	Link to the VIP's homepage
Source_code	Link to the VIP's source code

Publication

Year	The year the VIP was published
PubMed_ID	PubMed ID of the publication
Title	Title of the publication
DOI	Digital object identifier (DOI) of the publication
Latest_publication	Binary value indicating if this is the latest publication of the VIP

Citation

Year1993	Number of citations the VIP received in 1993
Year1994	Number of citations the VIP received in 1994
Year1995	Number of citations the VIP received in 1995
Year1996	Number of citations the VIP received in 1996
Year1997	Number of citations the VIP received in 1997
Year1998	Number of citations the VIP received in 1998
Year1999	Number of citations the VIP received in 1999
Year2000	Number of citations the VIP received in 2000
Year2001	Number of citations the VIP received in 2001
Year2002	Number of citations the VIP received in 2002
Year2003	Number of citations the VIP received in 2003
Year2004	Number of citations the VIP received in 2004
Year2005	Number of citations the VIP received in 2005
Year2006	Number of citations the VIP received in 2006
Year2007	Number of citations the VIP received in 2007
Year2008	Number of citations the VIP received in 2008
Year2009	Number of citations the VIP received in 2009
Year2010	Number of citations the VIP received in 2010
Year2011	Number of citations the VIP received in 2011
Year2012	Number of citations the VIP received in 2012
Year2013	Number of citations the VIP received in 2013
Year2014	Number of citations the VIP received in 2014
Year2015	Number of citations the VIP received in 2015
Year2016	Number of citations the VIP received in 2016
Year2017	Number of citations the VIP received in 2017
Year2018	Number of citations the VIP received in 2018
Year2019	Number of citations the VIP received in 2019
Year2020	Number of citations the VIP received in 2020
Year2021	Number of citations the VIP received in 2021
Year2022	Number of citations the VIP received in 2022
Year2023	Number of citations the VIP received in 2023
Year2024	Number of citations the VIP received in 2024 until August 9
Citation_total_2024	Total number of citations received by the VIP until August 9, 2024

Note: Question marks in the table indicate that there is no information available

License Disclaimer: Users should determine if each tool and its licensing are appropriate for their intended use. The license descriptions are provided for convenience, and we do not guarantee their accuracy.
